# Supplementary figures and images for: Protective impacts of household-based tuberculosis contact tracing are robust across endemic incidence levels and community contact patterns
Source: PLoS Comput Biol. 2021 Feb 8;17(2):e1008713. doi: 10.1371/journal.pcbi.1008713 (PMC7895355; doi:10.1371/journal.pcbi.1008713)

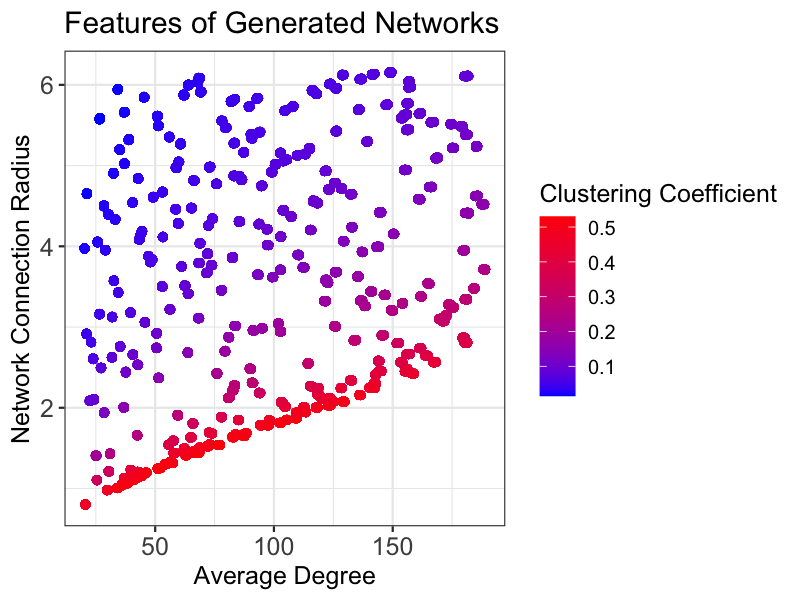

Supplement: S1 Fig — Community Average Degree by average connection radius colored by community global clustering coefficient. We generated a wide array of networks i.e., long range connections vs. short range clustered connections and/or many community contacts vs. few community contacts. The parameters we used to specify the networks (see Table 1) were different from the actual calculated metrics on the generated networks. The range of average degrees of generated networks was ∼20 to ∼190. The range of average connection radii of generated networks was from ∼0.8 to ∼6.2. (TIFF) [file pcbi.1008713.s001.tiff]

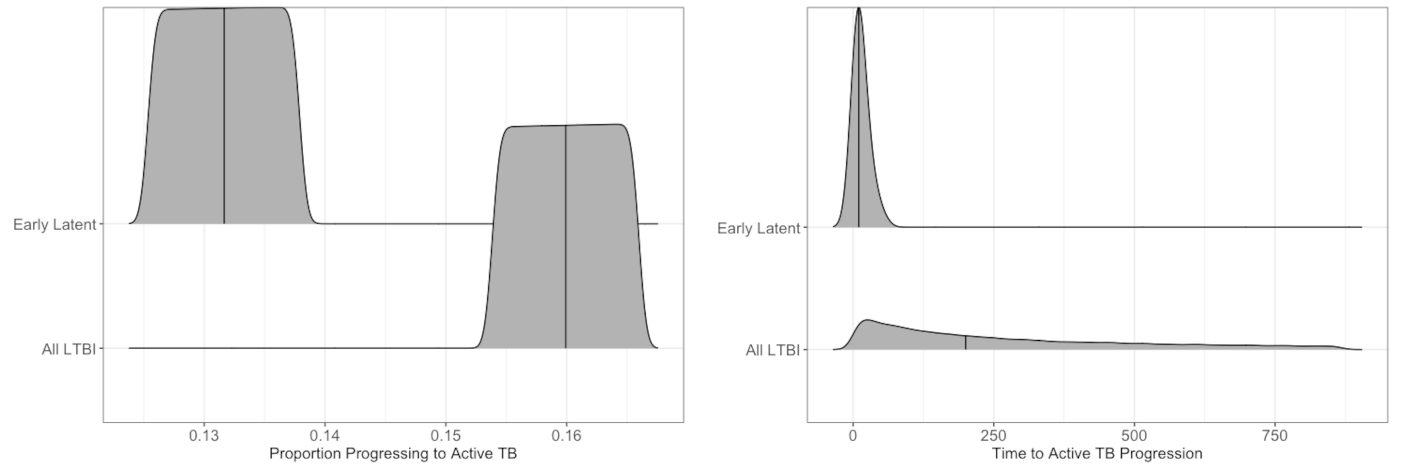

Supplement: S2 Fig — For all parameter sets, fraction progressing among those in the early latent state and across all latent TB states (left) and the time to progression for individuals in the early latent state and across all latent TB states (right). (TIF) [file pcbi.1008713.s002.tif]

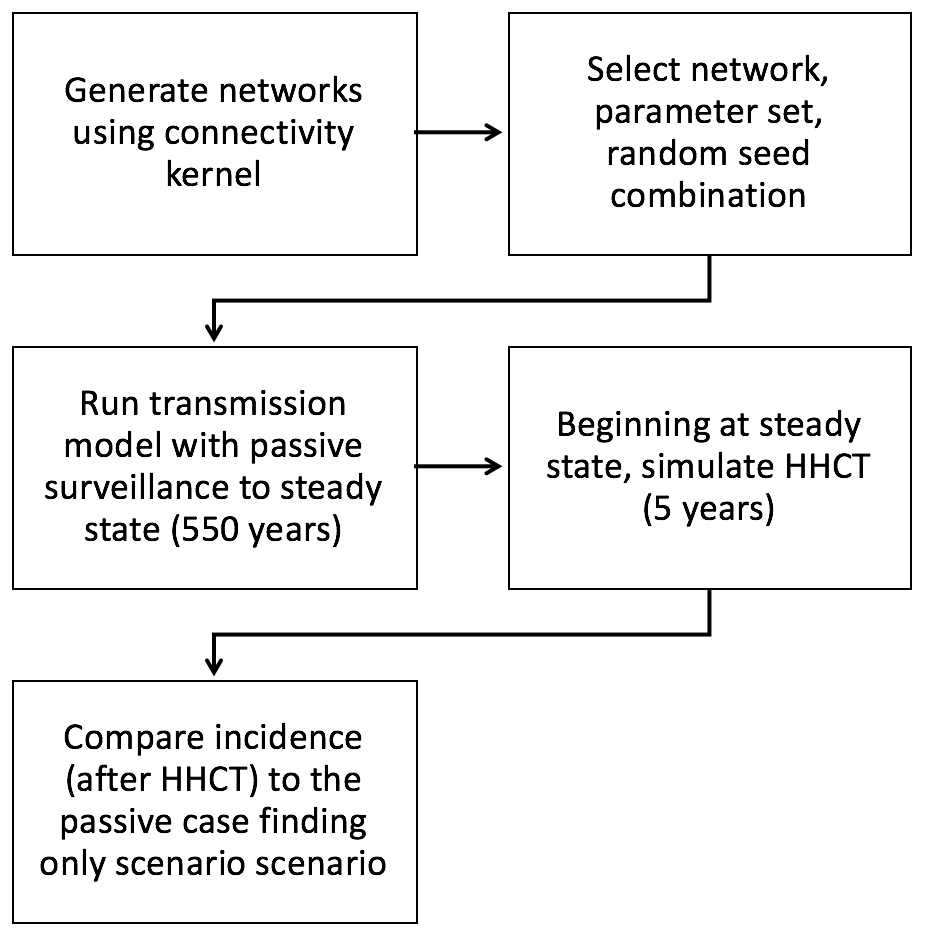

Supplement: S3 Fig — (TIFF) [file pcbi.1008713.s003.tiff]

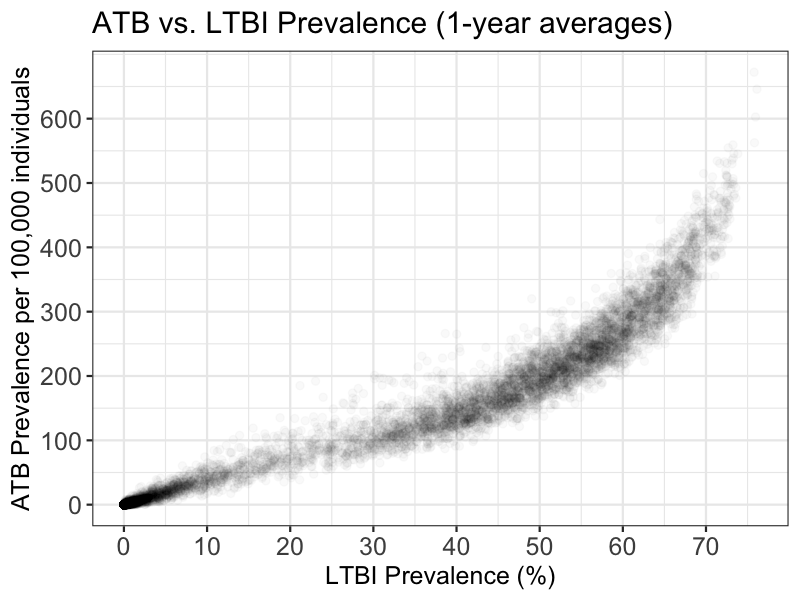

Supplement: S4 Fig — Active TB prevalence per 100,000 individuals vs. latent TB prevalence across all model runs in which no intervention (passive-detection only) was administered. Points correspond to monthly average values over the final year of the simulation. Latent levels were calculated by summing early latent and late latent states. (TIFF) [file pcbi.1008713.s004.tiff]

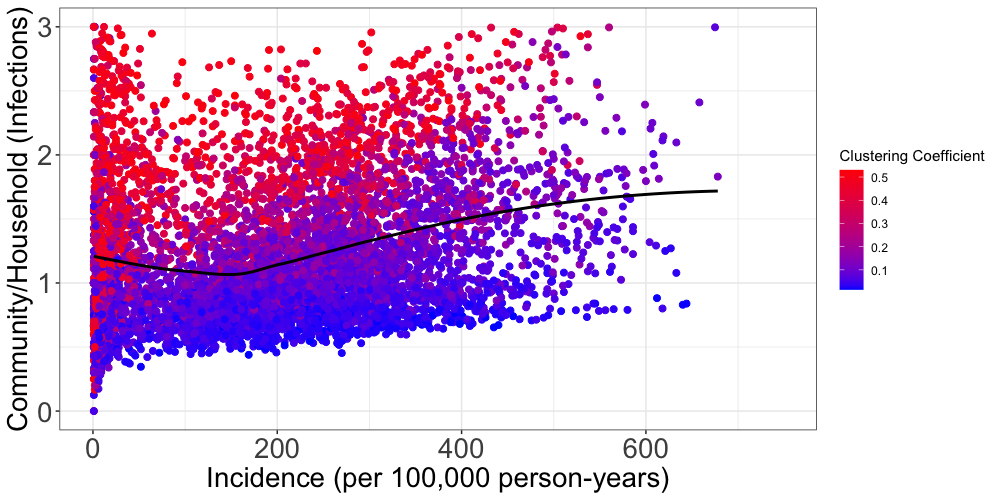

Supplement: S5 Fig — Annual number of TB infections (i.e., new cases of EL) attributed to community vs. household transmission (y-axis) and incidence levels immediately before ACF (x-axis). Points are colored by community clustering coefficient. These results are excluding model runs that did not result in an outbreak. The line is the fitted spline calculated using the LOESS method in R [33]. (TIFF) [file pcbi.1008713.s005.tiff]

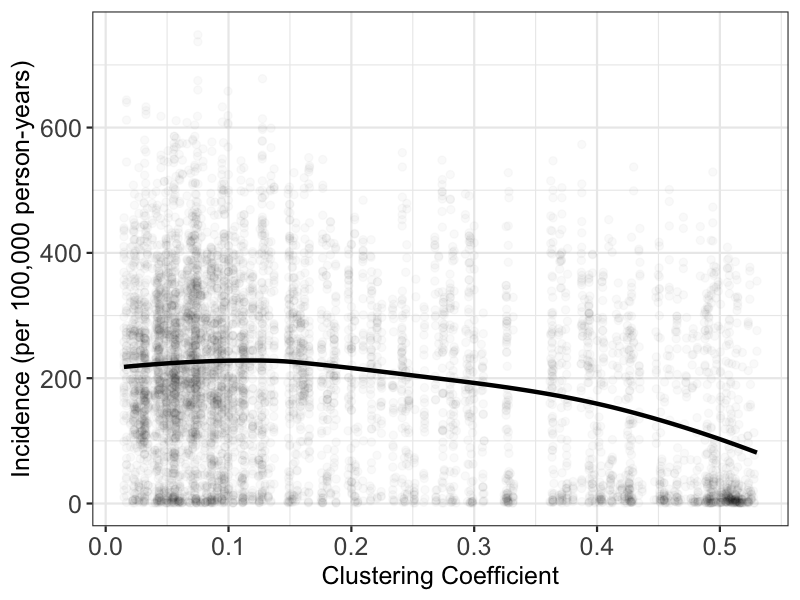

Supplement: S6 Fig — Active TB Incidence per 100,000 person-years vs. clustering coefficient. These results are excluding model runs that did not result in an outbreak. The line is the fitted spline calculated using the LOESS method in R [33]. (TIFF) [file pcbi.1008713.s006.tiff]

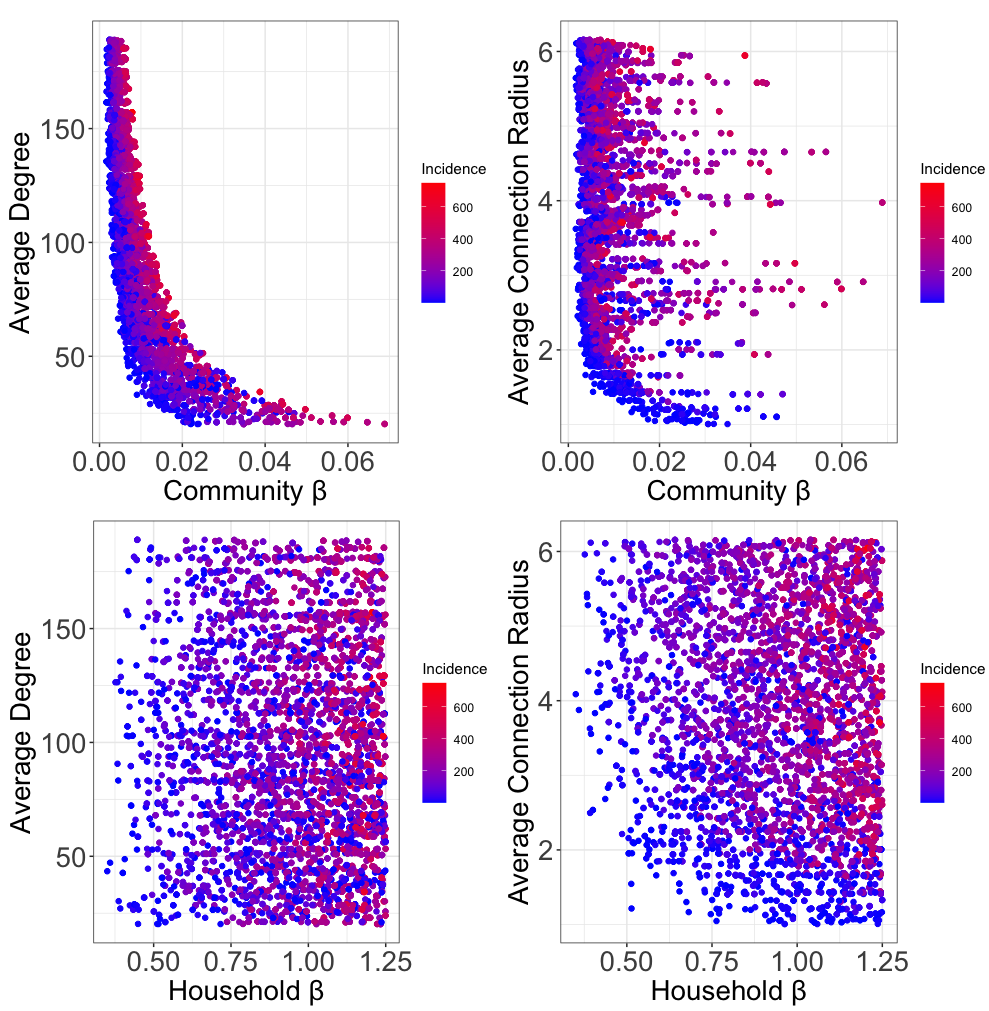

Supplement: S7 Fig — Community beta vs. average degree colored by incidence (top left). Community beta vs. average connection radius colored by incidence (top right). Household beta vs. average degree colored by incidence (bottom left). Household beta vs. average connection radius colored by incidence (bottom right). These results are excluding model runs that did not result in an outbreak. (TIF) [file pcbi.1008713.s007.tif]

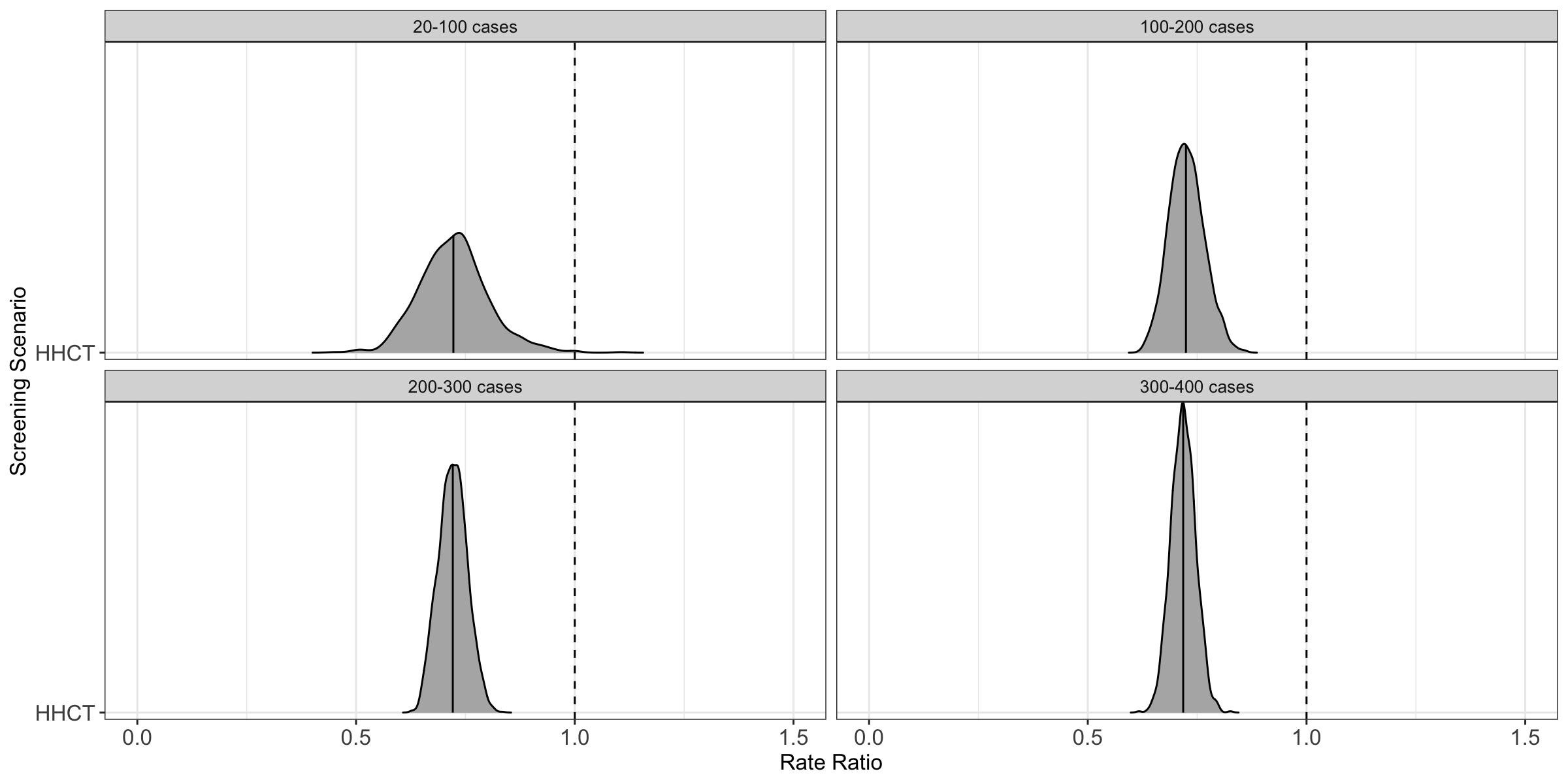

Supplement: S8 Fig — Ridgeline plot showing performance of HHCT across different pre-ACF incidence levels (per 100,000 person years) using passive detection only as a reference group. The median is denoted by the solid vertical black line and the dashed vertical black line denotes a null rate ratio (RR) equal to 1. (TIFF) [file pcbi.1008713.s008.tiff]

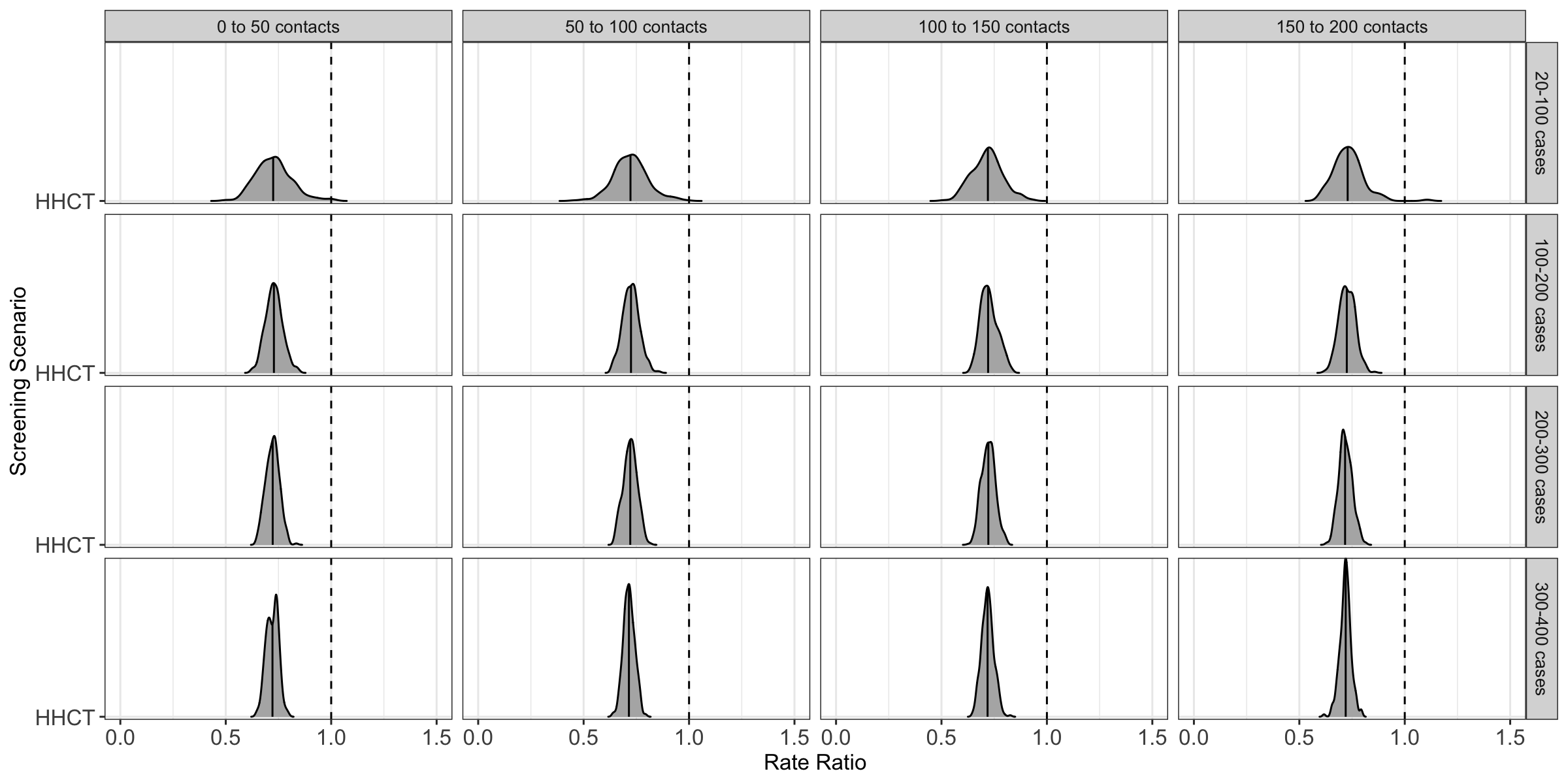

Supplement: S9 Fig — Ridgeline plot showing performance of screening interventions within strata of average degree and incidence (per 100,000 person years). The median is denoted by the solid vertical black line. The dashed vertical black line denotes a null RR equal to 1. (TIFF) [file pcbi.1008713.s009.tiff]

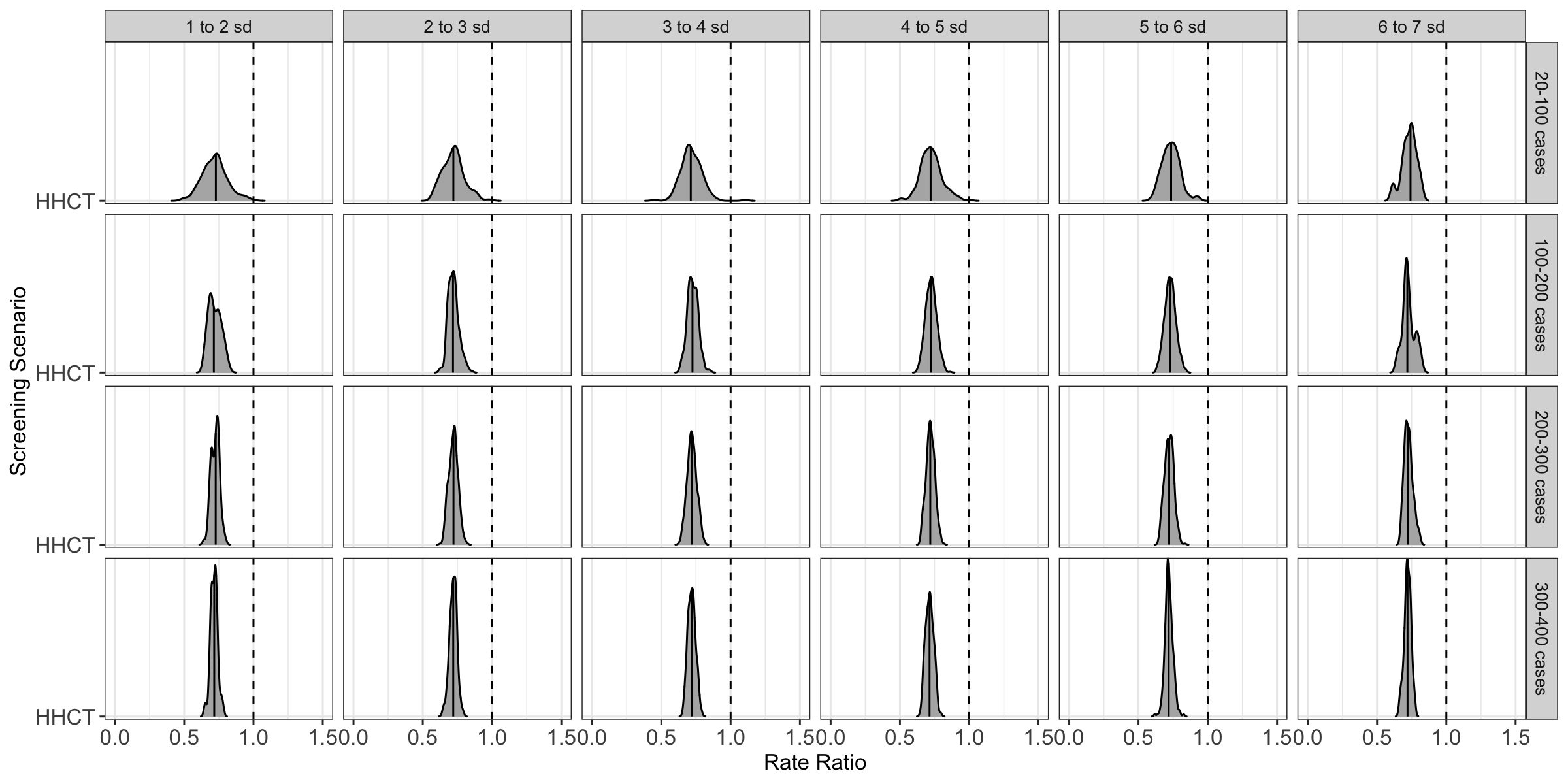

Supplement: S10 Fig — Ridgeline plot showing performance of screening interventions within strata of average connection radius (σ) and incidence (per 100,000 person years). The median is denoted by the solid vertical black line. The dashed vertical black line denotes a null RR equal to 1. (TIFF) [file pcbi.1008713.s010.tiff]

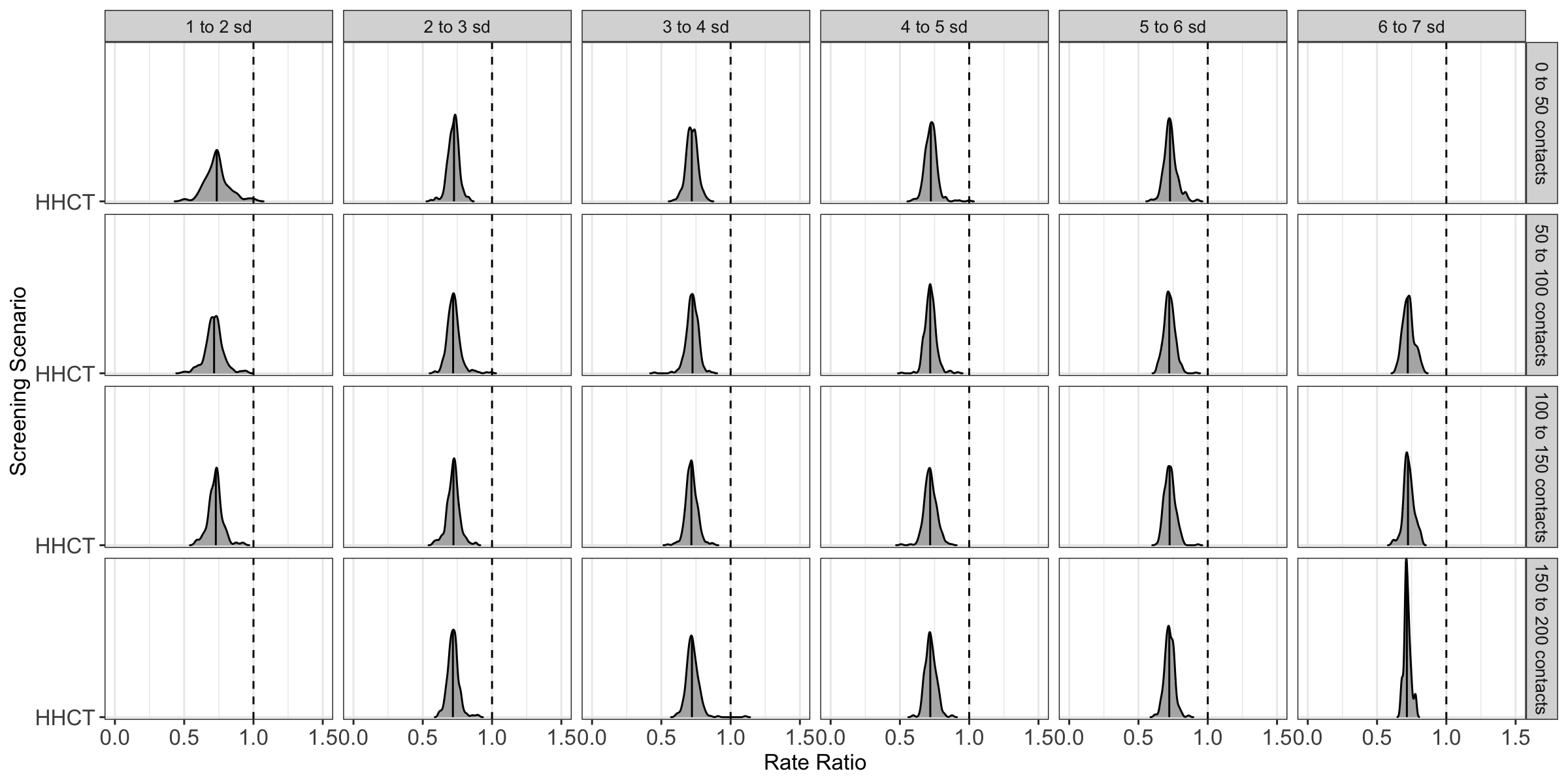

Supplement: S11 Fig — Ridgeline plot showing performance of screening interventions within strata of average degree and average connection radius (σ). The median is denoted by the solid vertical black line. The dashed vertical black line denotes a null RR equal to 1. (TIFF) [file pcbi.1008713.s011.tiff]

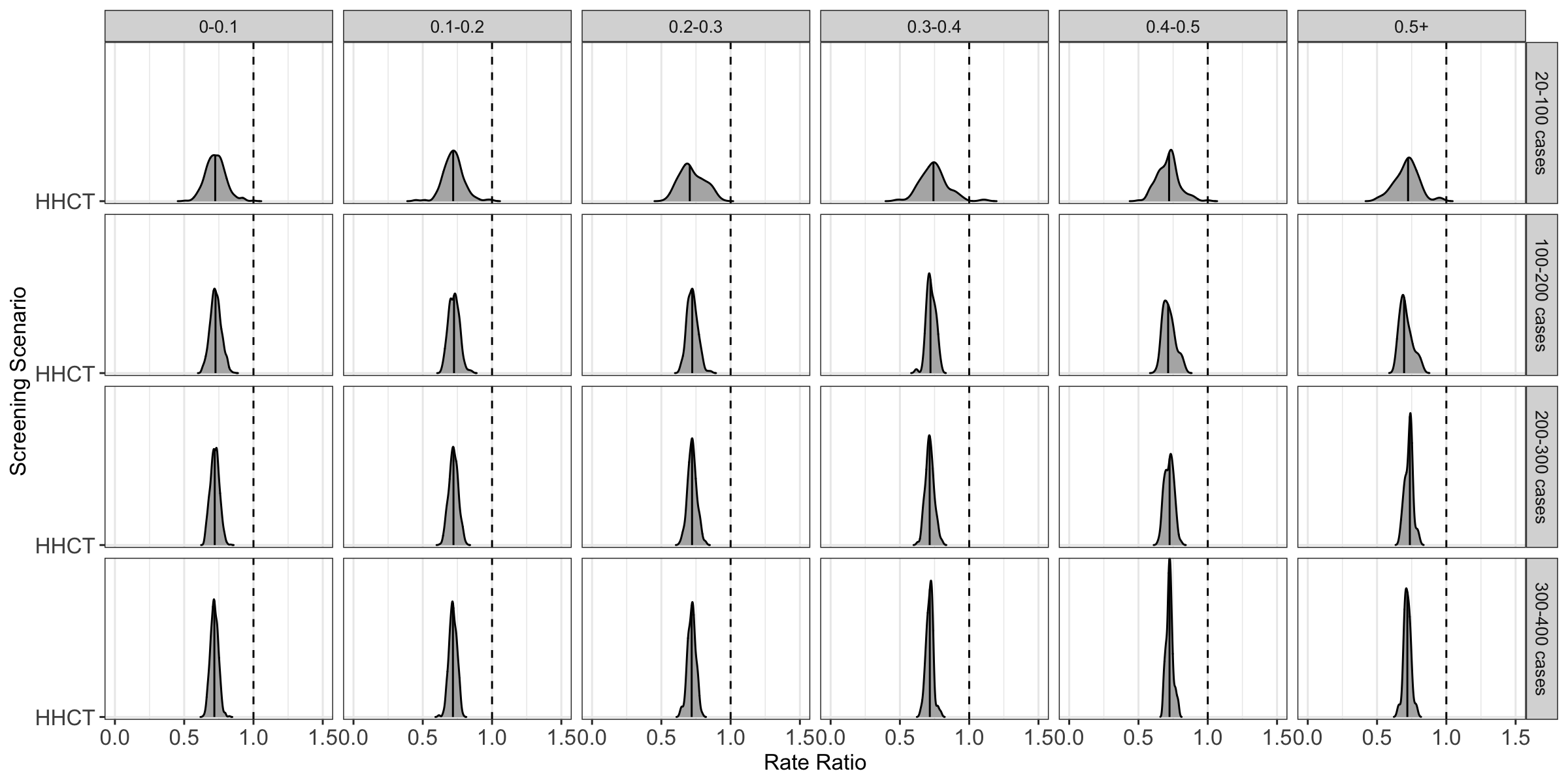

Supplement: S12 Fig — Ridgeline plot showing performance of screening interventions within strata of community clustering coefficient and incidence level. The median is denoted by the solid vertical black line. The dashed vertical black line denotes a null RR equal to 1. (TIFF) [file pcbi.1008713.s012.tiff]

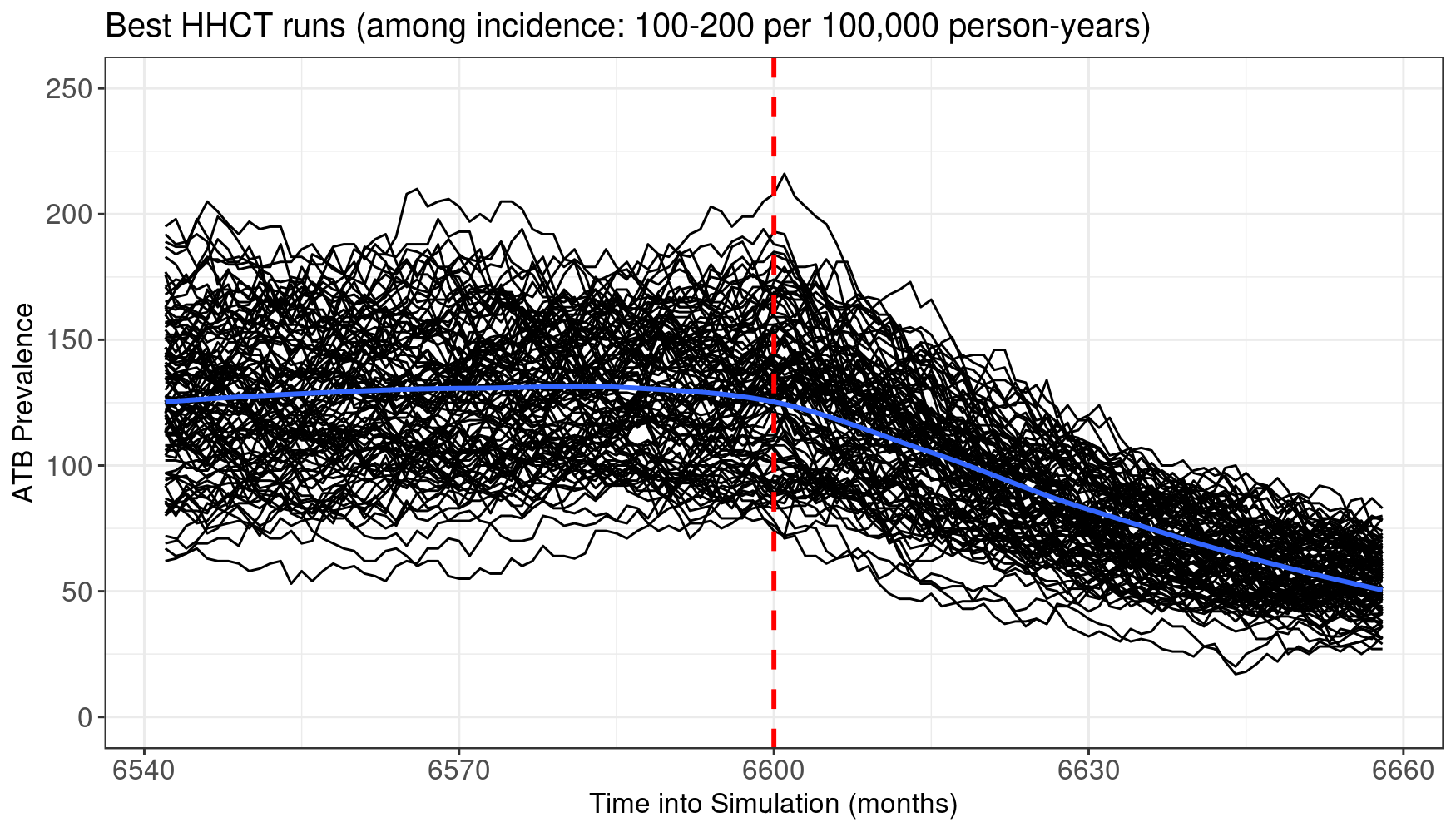

Supplement: S13 Fig — The 100 best performing model runs (i.e., with the lowest rate ratios), for HHCT and among incidence rates between 100 to 200 cases per 100,000 person years. We plotted these trajectories immediately before and after ACF was implemented. The vertical dashed red line indicates the time step in which active screening interventions were implemented. The line is the fitted spline calculated using the LOESS method in R [33]. (TIFF) [file pcbi.1008713.s013.tiff]

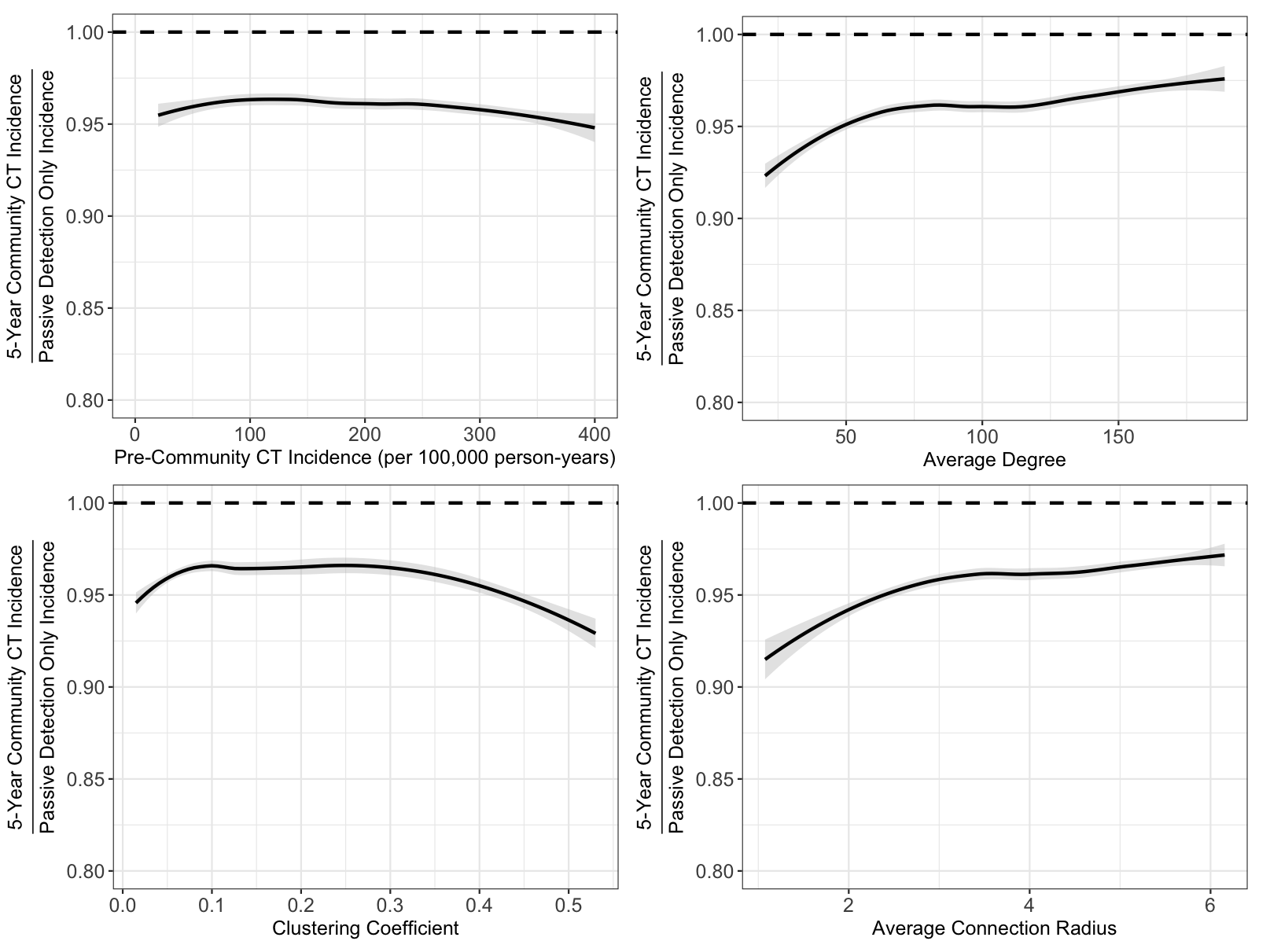

Supplement: S14 Fig — Fitted splines representing relationship between all RRs comparing community CT to passive surveillance only and (1) the incidence rate immediately before community CT (per 100,000 person-years) (top left), (2) the average degree (top right), (3) the community clustering coefficient (bottom left), and (4) the average connection radius (bottom right). Lines are splines calculated using the LOESS method in R [33]. Among model runs with incidence rates between 20 and 400 cases per 100,000 person-years. Shaded regions represent 95% confidence intervals. (TIF) [file pcbi.1008713.s014.tif]

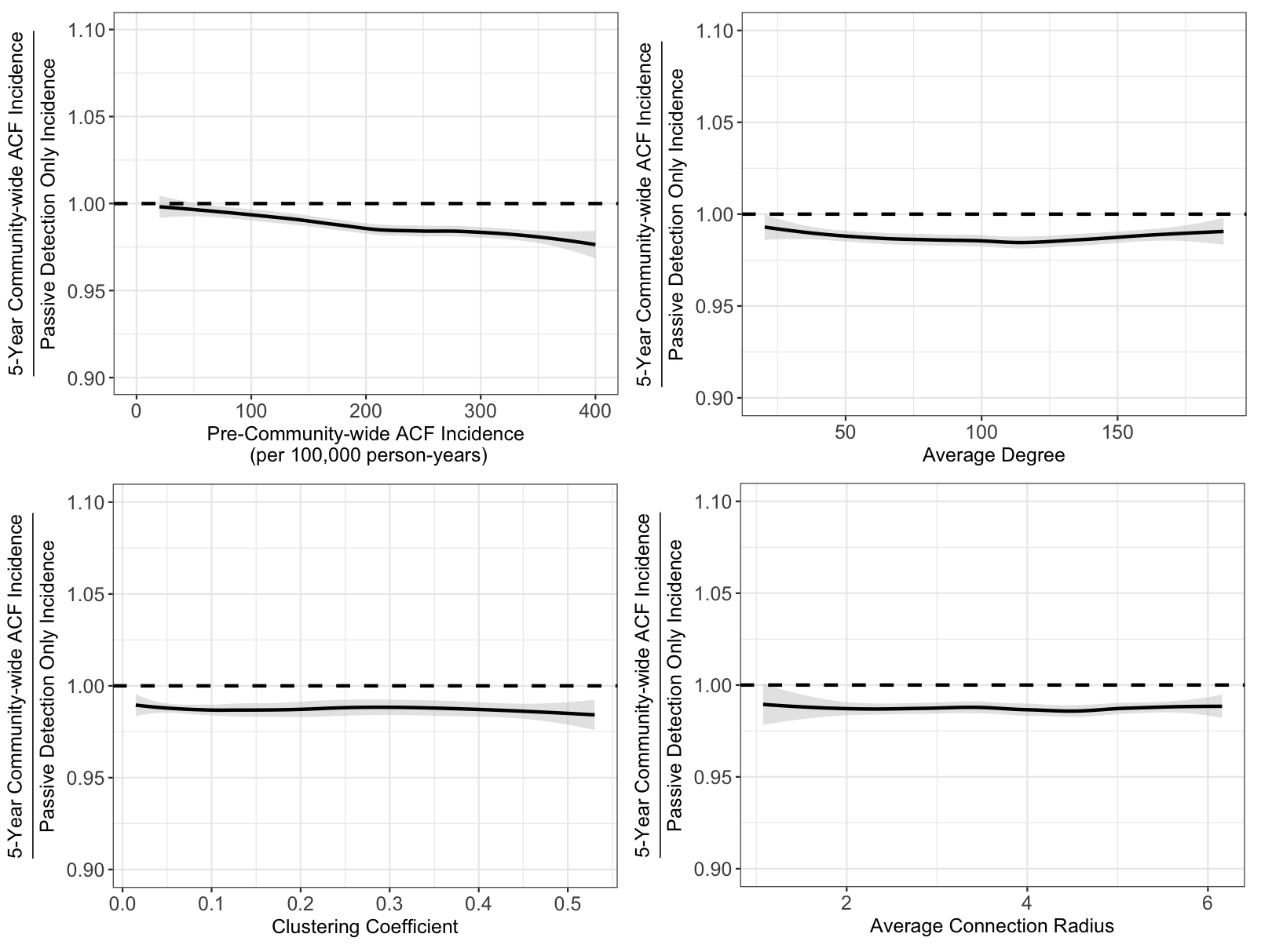

Supplement: S15 Fig — Fitted splines representing relationship between all RRs comparing community-wide ACF to passive surveillance only and (1) the incidence rate immediately before community-wide ACF (per 100,000 person-years) (top left), (2) the average degree (top right), (3) the community clustering coefficient (bottom left), and (4) the average connection radius (bottom right). Lines are splines calculated using the LOESS method in R [33]. Among model runs with incidence rates between 20 and 400 cases per 100,000 person-years. Shaded regions represent 95% confidence intervals. (TIF) [file pcbi.1008713.s015.tif]

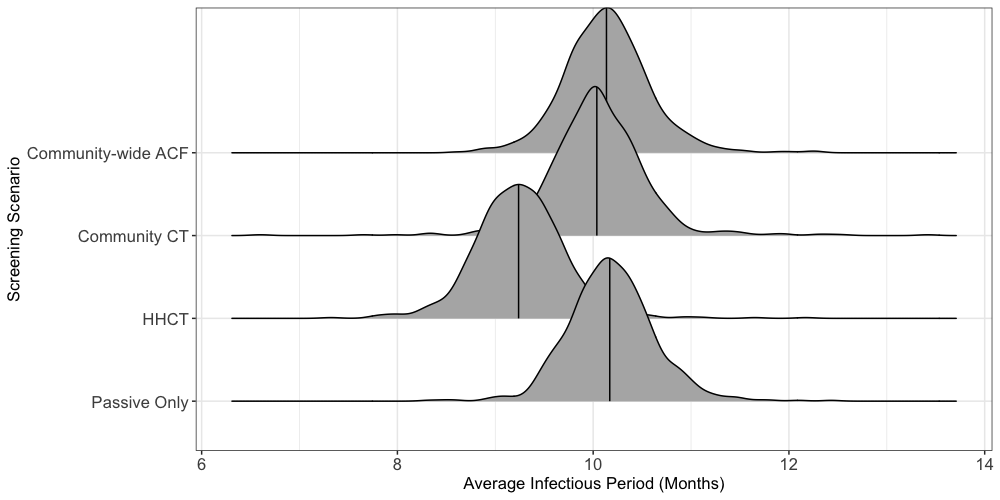

Supplement: S16 Fig — Ridgeline plot showing how the average infectious period in months varies by screening intervention. The median is denoted by the solid vertical black line. (TIFF) [file pcbi.1008713.s016.tiff]

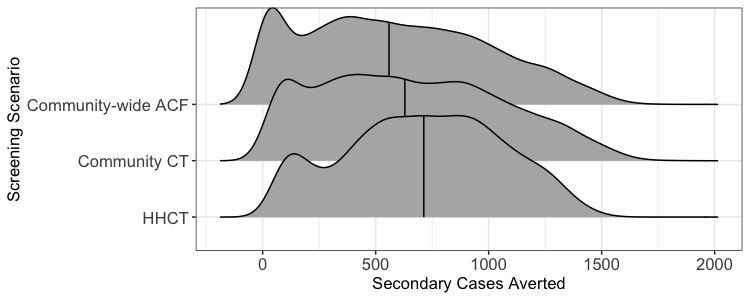

Supplement: S17 Fig — Ridgeline plot showing how the average number of secondary cases averted among household contacts varies by screening intervention. The median is denoted by the solid vertical black line. (TIFF) [file pcbi.1008713.s017.tiff]

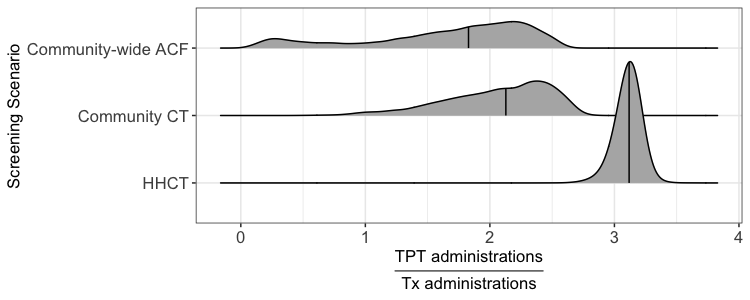

Supplement: S18 Fig — Ridgeline plot showing the total number of preventive therapy administrations divided by the total number of treatment administrations by screening intervention. The median is denoted by the solid vertical black line. (TIFF) [file pcbi.1008713.s018.tiff]

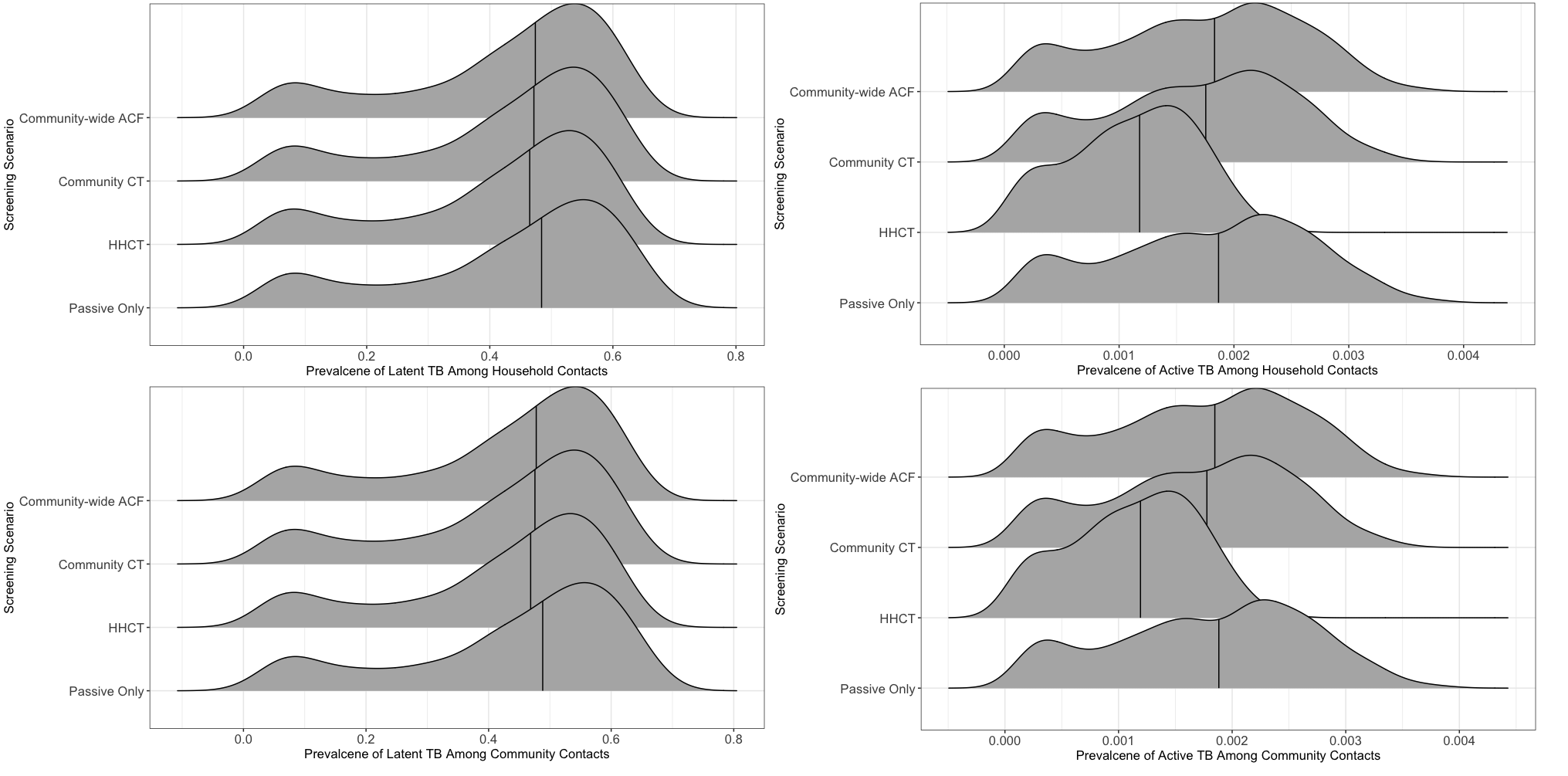

Supplement: S19 Fig — Ridegeline plots representing the prevalence of LTBI and active TB among household and community contacts across all simulation runs. (1) LTBI among household contacts (top left), (2) Active TB among household contacts (top right), (3) LTBI among community contacts (bottom left), and (4) Active TB among community contacts (bottom right). The median is denoted by the solid vertical black line. (TIF) [file pcbi.1008713.s019.tif]

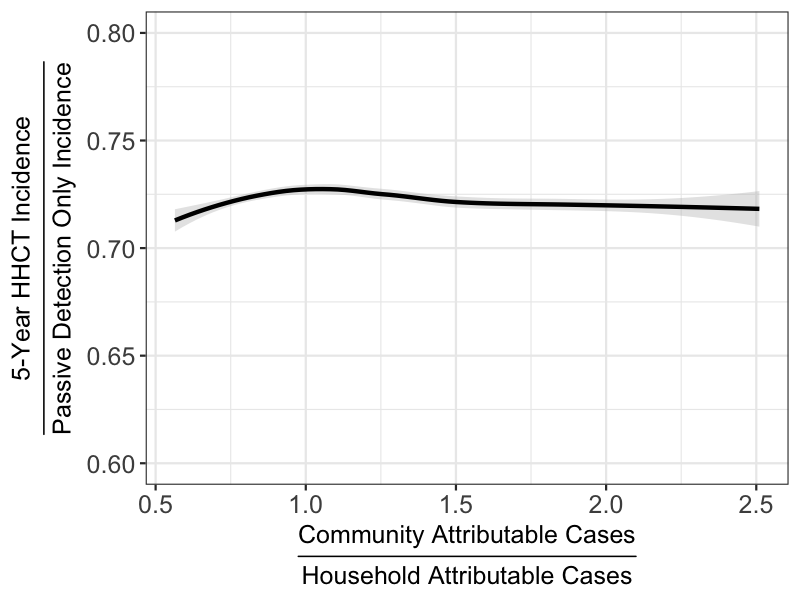

Supplement: S20 Fig — Fitted splines representing relationship between all RRs comparing HHCT to passive surveillance only and the number of community attributable infections to household attributable infections. We removed extreme community to household transmission ratios <2.5% and >97.5% to make the figure easier to interpret. Lines are splines calculated using the LOESS method in R [33]. Among model runs with incidence rates between 20 and 400 cases per 100,000 person-years. Shaded regions represent 95% confidence intervals. (TIFF) [file pcbi.1008713.s020.tiff]

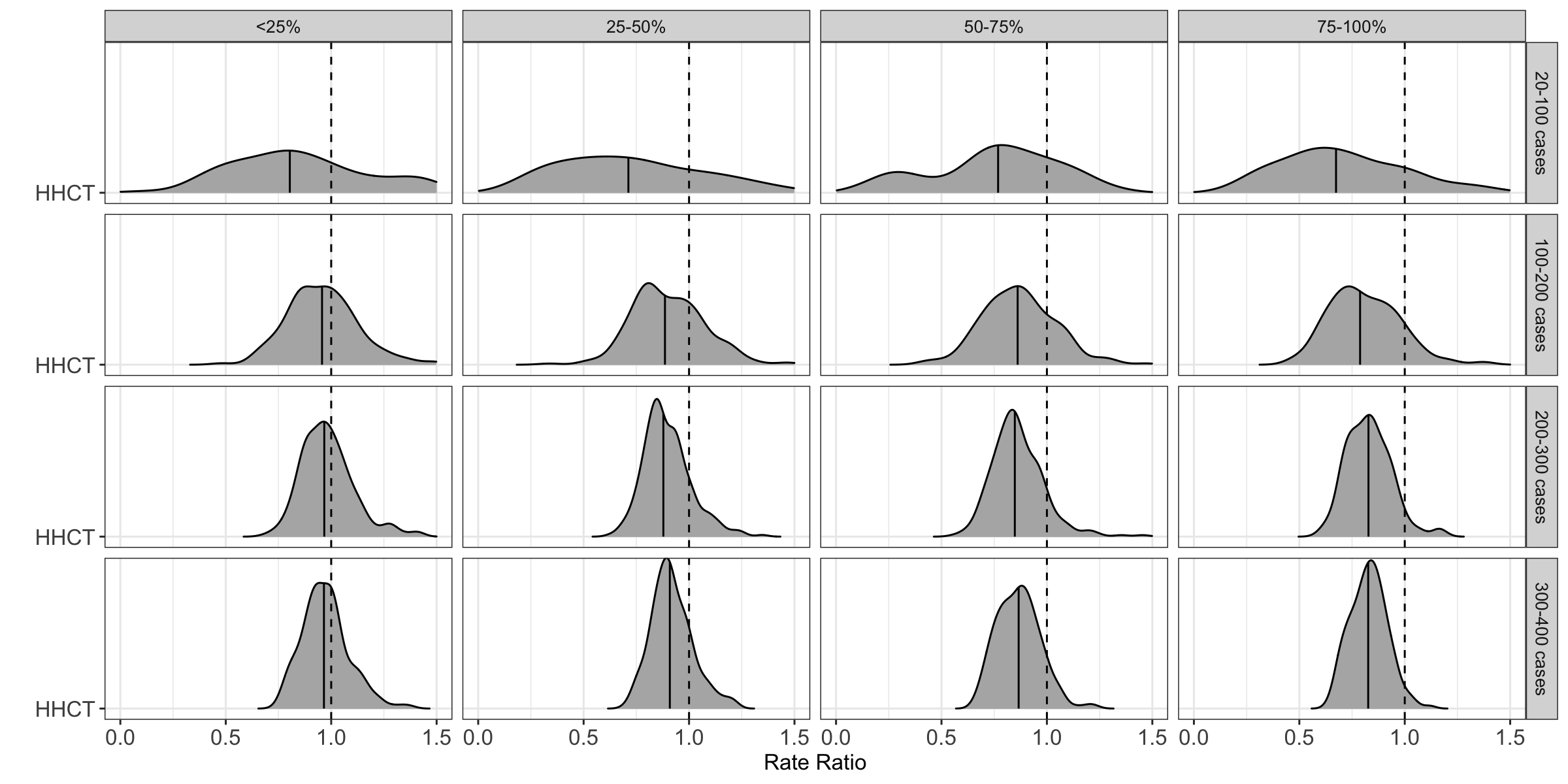

Supplement: S21 Fig — Ridgeline plot showing performance of screening interventions within strata of HHCT coverage and incidence level. The median is denoted by the solid vertical black line. The dashed vertical black line denotes a null RR equal to 1. (TIFF) [file pcbi.1008713.s021.tiff]

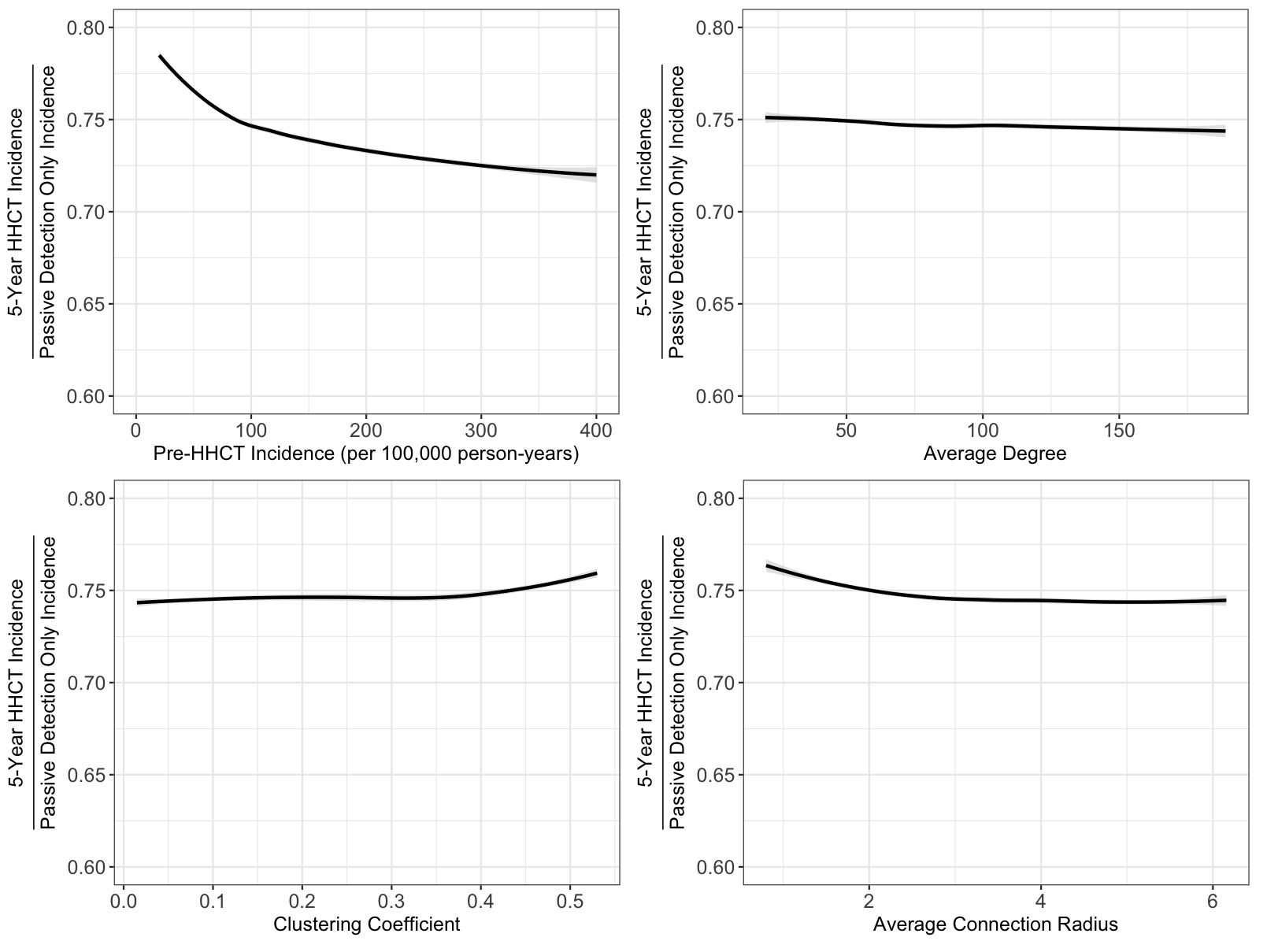

Supplement: S22 Fig — Fitted splines representing relationship between all RRs comparing HHCT to passive surveillance only and (1) the incidence rate immediately before HHCT (per 100,000 person-years) (top left), (2) the average degree (top right), (3) the community clustering coefficient (bottom left), and (4) the average connection radius (bottom right). Lines are splines calculated using the LOESS method in R [33]. Among model runs with incidence rates between 20 and 400 cases per 100,000 person-years. Shaded regions represent 95% confidence intervals. (TIF) [file pcbi.1008713.s022.tif]

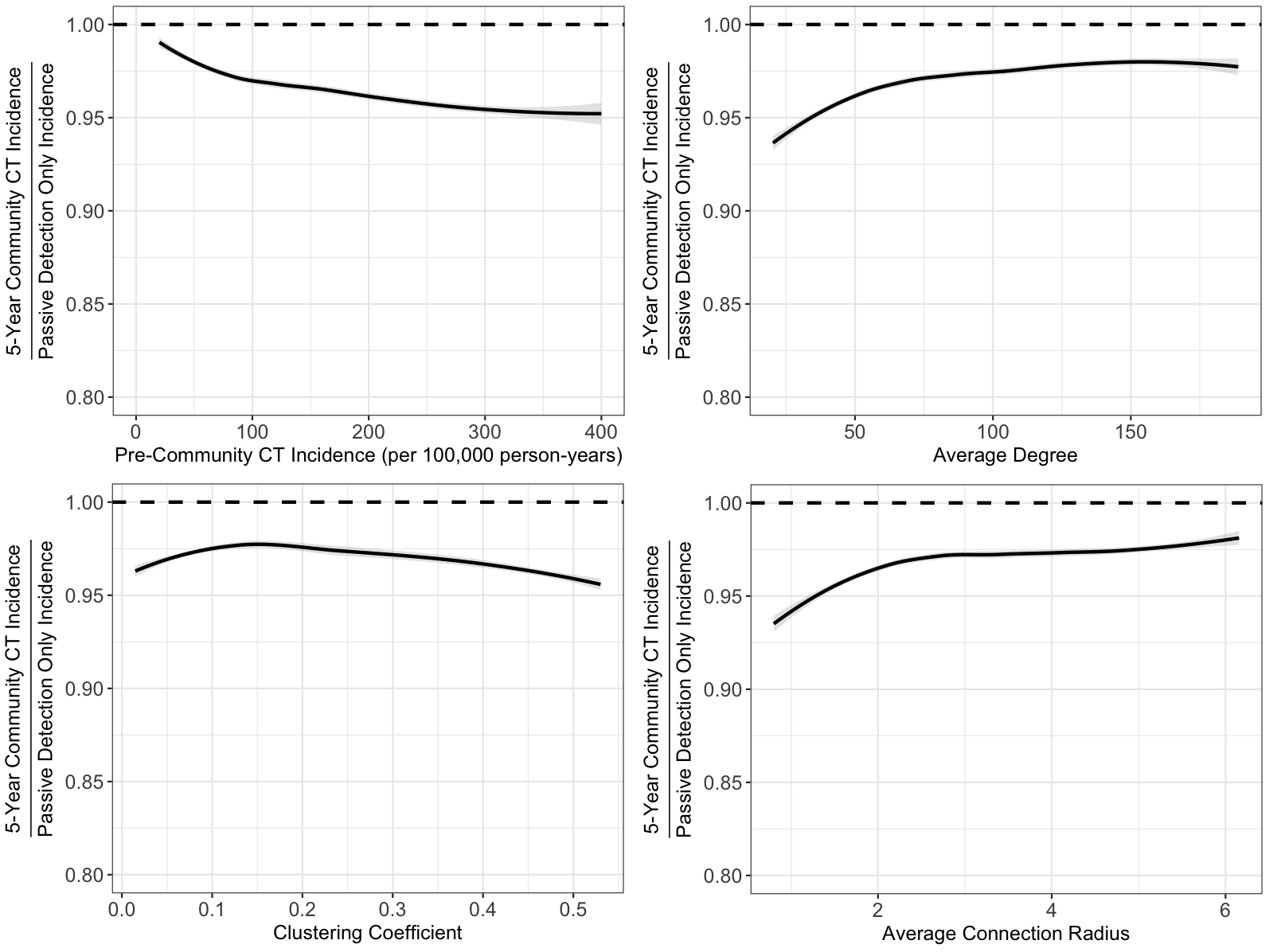

Supplement: S23 Fig — Fitted splines representing relationship between all RRs comparing Community CT to passive surveillance only and (1) the incidence rate immediately before community CT (per 100,000 person-years) (top left), (2) the average degree (top right), (3) the community clustering coefficient (bottom left), and (4) the average connection radius (bottom right). Lines are splines calculated using the LOESS method in R [33]. Among model runs with incidence rates between 20 and 400 cases per 100,000 person-years. Shaded regions represent 95% confidence intervals. (TIF) [file pcbi.1008713.s023.tif]

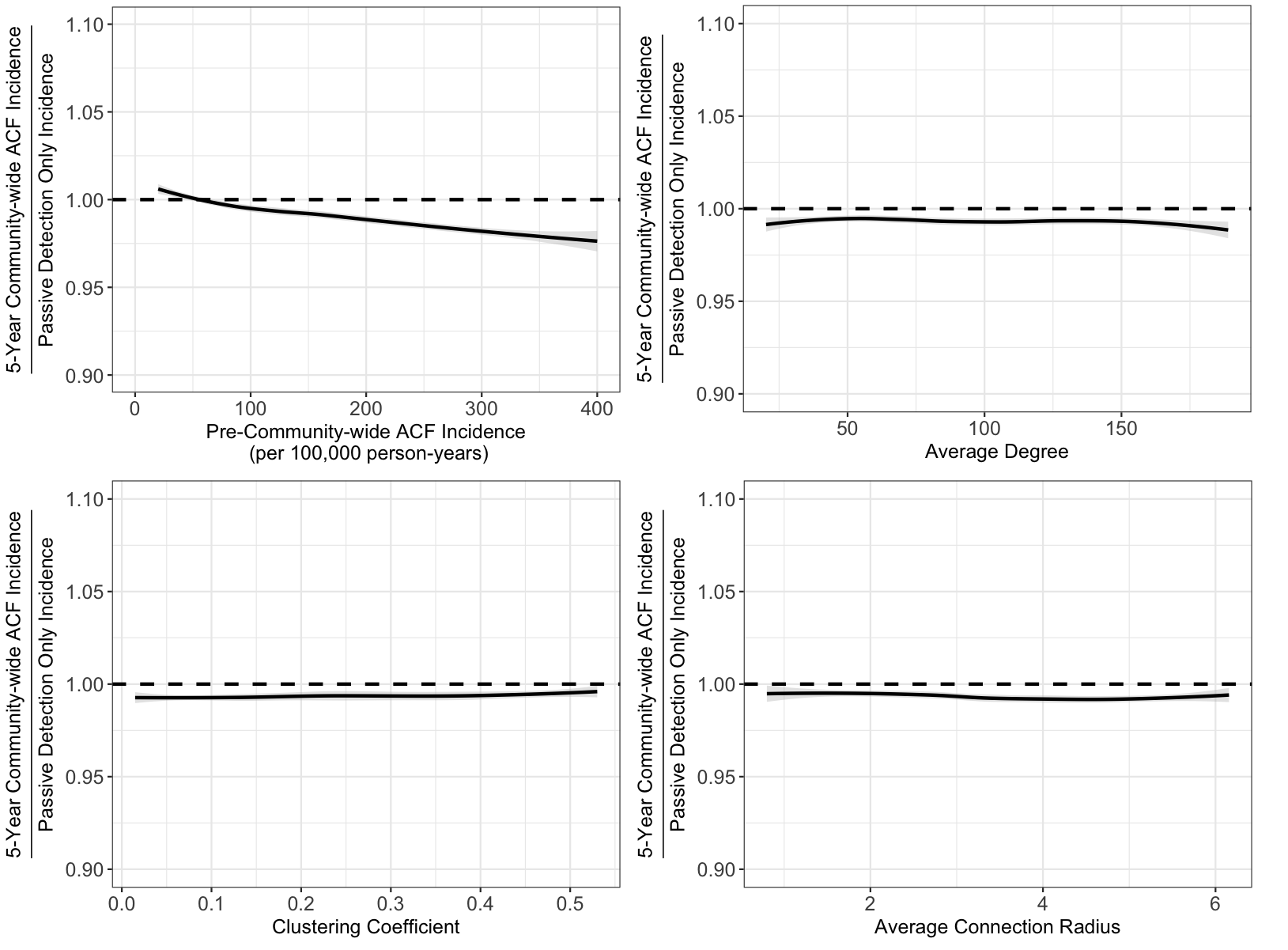

Supplement: S24 Fig — Fitted splines representing relationship between all RRs comparing Community-wide ACF to passive surveillance only and (1) the incidence rate immediately before Community-wide ACF (per 100,000 person-years) (top left), (2) the average degree (top right), (3) the community clustering coefficient (bottom left), and (4) the average connection radius (bottom right). Lines are splines calculated using the LOESS method in R [33]. Among model runs with incidence rates between 20 and 400 cases per 100,000 person-years. Shaded regions represent 95% confidence intervals. (TIF) [file pcbi.1008713.s024.tif]
