# Supplementary material for: Protective impacts of household-based tuberculosis contact tracing are robust across endemic incidence levels and community contact patterns
Source: PLoS Comput Biol. 2021 Feb 8;17(2):e1008713. doi: 10.1371/journal.pcbi.1008713 (PMC7895355; doi:10.1371/journal.pcbi.1008713)
Supplement: S1 Table — (PDF) [file pcbi.1008713.s025.pdf]

S1 Table: HHCT RRs by Incidence Strata in Order of Performance.

**Incidence Level (per 100,000 person-years)**	**Mean RR**	**Mean RR (SD)**	**Number of Runs**
300-400 cases	0.72	0.03	1039
200-300 cases	0.72	0.03	1704
20-100 cases	0.72	0.08	869
100-200 cases	0.73	0.04	1559
