# Supplementary material for: Protective impacts of household-based tuberculosis contact tracing are robust across endemic incidence levels and community contact patterns
Source: PLoS Comput Biol. 2021 Feb 8;17(2):e1008713. doi: 10.1371/journal.pcbi.1008713 (PMC7895355; doi:10.1371/journal.pcbi.1008713)
Supplement: S2 Table — (PDF) [file pcbi.1008713.s026.pdf]

S2 Table: HHCT RRs by Average Degree Strata in Order of Performance

Average Degree (contacts)	Mean RR	Mean RR (SD)	Number of Runs
50 to 100 contacts	0.72	0.05	1819
100 to 150 contacts	0.72	0.04	1475
150 to 200 contacts	0.72	0.04	996
0 to 50 contacts	0.72	0.05	881
