# Supplementary material for: Protective impacts of household-based tuberculosis contact tracing are robust across endemic incidence levels and community contact patterns
Source: PLoS Comput Biol. 2021 Feb 8;17(2):e1008713. doi: 10.1371/journal.pcbi.1008713 (PMC7895355; doi:10.1371/journal.pcbi.1008713)
Supplement: S3 Table — (PDF) [file pcbi.1008713.s027.pdf]

**S3 Table: HHCT RRs by Average Connection Radius in Order of Performance**

| Average Connection Radius | Mean RR | Mean RR (SD) | Number of Runs |
|---|---|---|---|
| 1 to 2 sd | 0.72 | 0.07 | 397 |
| 2 to 3 sd | 0.72 | 0.05 | 1065 |
| 3 to 4 sd | 0.72 | 0.04 | 1127 |
| 4 to 5 sd | 0.72 | 0.04 | 1167 |
| 5 to 6 sd | 0.73 | 0.04 | 1204 |
| 6 to 7 sd | 0.73 | 0.04 | 211 |
