# Supplementary material for: Protective impacts of household-based tuberculosis contact tracing are robust across endemic incidence levels and community contact patterns
Source: PLoS Comput Biol. 2021 Feb 8;17(2):e1008713. doi: 10.1371/journal.pcbi.1008713 (PMC7895355; doi:10.1371/journal.pcbi.1008713)
Supplement: S4 Table — (PDF) [file pcbi.1008713.s028.pdf]

S4 Table: HHCT RRs by Clustering Coefficient Strata in Order of Performance

**Clustering Coefficient**	**Mean RR**	**Mean RR (SD)**	**Number of Runs**
0.5+	0.72	0.06	156
0.4-0.5	0.72	0.06	448
0.1-0.2	0.72	0.04	1037
0.2-0.3	0.72	0.05	488
0-0.1	0.72	0.04	2723
0.3-0.4	0.72	0.06	319
