# Supplementary material for: Protective impacts of household-based tuberculosis contact tracing are robust across endemic incidence levels and community contact patterns
Source: PLoS Comput Biol. 2021 Feb 8;17(2):e1008713. doi: 10.1371/journal.pcbi.1008713 (PMC7895355; doi:10.1371/journal.pcbi.1008713)
Supplement: S5 Table — (PDF) [file pcbi.1008713.s029.pdf]

**S5 Table: Community CT compared with Passive Surveillance only RRs by Incidence Strata in Order of Performance.**

| **Incidence Level (per 100,000 person-years)** | **Mean RR** | **Mean RR (SD)** | **Number of Runs** |
|---|---|---|---|
| 300-400 cases | 0.95 | 0.04 | 1039 |
| 20-100 cases | 0.96 | 0.1 | 869 |
| 200-300 cases | 0.96 | 0.04 | 1704 |
| 100-200 cases | 0.96 | 0.06 | 1559 |
