# Supplementary material for: Protective impacts of household-based tuberculosis contact tracing are robust across endemic incidence levels and community contact patterns
Source: PLoS Comput Biol. 2021 Feb 8;17(2):e1008713. doi: 10.1371/journal.pcbi.1008713 (PMC7895355; doi:10.1371/journal.pcbi.1008713)
Supplement: S6 Table — (PDF) [file pcbi.1008713.s030.pdf]

**S6 Table: Community CT compared with Passive Surveillance Only RRs by Average Degree Strata in Order of Performance**

| Average Degree (contacts) | Mean RR | Mean RR (SD) | Number of Runs |
|---|---|---|---|
| 0 to 50 contacts | 0.94 | 0.06 | 881 |
| 50 to 100 contacts | 0.96 | 0.06 | 1819 |
| 100 to 150 contacts | 0.96 | 0.06 | 1475 |
| 150 to 200 contacts | 0.97 | 0.06 | 996 |
