# Supplementary material for: Protective impacts of household-based tuberculosis contact tracing are robust across endemic incidence levels and community contact patterns
Source: PLoS Comput Biol. 2021 Feb 8;17(2):e1008713. doi: 10.1371/journal.pcbi.1008713 (PMC7895355; doi:10.1371/journal.pcbi.1008713)
Supplement: S7 Table — (PDF) [file pcbi.1008713.s031.pdf]

S7 Table: Community CT compared with Passive Surveillance Only RRs by Average Connection Radius in Order of Performance

Average Connection Radius	Mean RR	Mean RR (SD)	Number of Runs
1 to 2 sd	0.94	0.08	397
2 to 3 sd	0.95	0.06	1065
3 to 4 sd	0.96	0.06	1127
4 to 5 sd	0.96	0.06	1167
5 to 6 sd	0.97	0.05	1204
6 to 7 sd	0.97	0.05	211
