# Supplementary material for: Protective impacts of household-based tuberculosis contact tracing are robust across endemic incidence levels and community contact patterns
Source: PLoS Comput Biol. 2021 Feb 8;17(2):e1008713. doi: 10.1371/journal.pcbi.1008713 (PMC7895355; doi:10.1371/journal.pcbi.1008713)
Supplement: S8 Table — (PDF) [file pcbi.1008713.s032.pdf]

S8 Table: Community CT compared with Passive Surveillance Only RRs by Clustering Coefficient Strata in Order of Performance

Clustering Coefficient	Mean RR	Mean RR (SD)	Number of Runs
0.5+	0.94	0.08	156
0.4-0.5	0.94	0.07	448
0.3-0.4	0.96	0.07	319
0-0.1	0.96	0.06	2723
0.1-0.2	0.96	0.06	1037
0.2-0.3	0.97	0.06	488
