# Supplementary material for: Protective impacts of household-based tuberculosis contact tracing are robust across endemic incidence levels and community contact patterns
Source: PLoS Comput Biol. 2021 Feb 8;17(2):e1008713. doi: 10.1371/journal.pcbi.1008713 (PMC7895355; doi:10.1371/journal.pcbi.1008713)
Supplement: S9 Table — (PDF) [file pcbi.1008713.s033.pdf]

S9 Table: Community-wide ACF compared with Passive Surveillance Only RRs by Incidence Strata in Order of Performance.

Incidence Level (per 100,000 person-years)	Mean RR	Mean RR (SD)	Number of Runs
300-400 cases	0.98	0.04	1039
200-300 cases	0.98	0.04	1704
100-200 cases	0.99	0.06	1559
20-100 cases	1	0.11	869
