# Supplementary material for: Protective impacts of household-based tuberculosis contact tracing are robust across endemic incidence levels and community contact patterns
Source: PLoS Comput Biol. 2021 Feb 8;17(2):e1008713. doi: 10.1371/journal.pcbi.1008713 (PMC7895355; doi:10.1371/journal.pcbi.1008713)
Supplement: S10 Table — (PDF) [file pcbi.1008713.s034.pdf]

S10 Table: Community-wide ACF compared with Passive Surveillance Only RRs by Average Degree Strata in Order of Performance

**Average Degree (contacts)**	**Mean RR**	**Mean RR (SD)**	**Number of Runs**
100 to 150 contacts	0.98	0.06	1475
50 to 100 contacts	0.99	0.06	1819
150 to 200 contacts	0.99	0.06	996
0 to 50 contacts	0.99	0.06	881
