# Supplementary material for: Protective impacts of household-based tuberculosis contact tracing are robust across endemic incidence levels and community contact patterns
Source: PLoS Comput Biol. 2021 Feb 8;17(2):e1008713. doi: 10.1371/journal.pcbi.1008713 (PMC7895355; doi:10.1371/journal.pcbi.1008713)
Supplement: S11 Table — (PDF) [file pcbi.1008713.s035.pdf]

S11 Table: Community-wide ACF compared with Passive Surveillance Only RRs by Average Connection Radius in Order of Performance

Average Connection Radius	Mean RR	Mean RR (SD)	Number of Runs
6 to 7 sd	0.99	0.05	211
4 to 5 sd	0.99	0.06	1167
2 to 3 sd	0.99	0.07	1065
3 to 4 sd	0.99	0.06	1127
1 to 2 sd	0.99	0.09	397
5 to 6 sd	0.99	0.05	1204
