# Supplementary material for: Protective impacts of household-based tuberculosis contact tracing are robust across endemic incidence levels and community contact patterns
Source: PLoS Comput Biol. 2021 Feb 8;17(2):e1008713. doi: 10.1371/journal.pcbi.1008713 (PMC7895355; doi:10.1371/journal.pcbi.1008713)
Supplement: S13 Table — Exploring the impacts of stochasticity, network realization and model parameterization on RRs. (PDF) [file pcbi.1008713.s037.pdf]

S13 Table: Exploring the impacts of stochasticity, network realization and model parameterization on RRs.

**Parameters**	**Coefficients (SE)**
Num. obs.	15513
Num. groups: network realization	1588
(Intercept)	1.02 (0.03)***
Infection C	$-7e^{-6}$ ($3.4e^{-6}$)*
HHCT	-0.12 (0.04)***
Community CT	-0.05 (0.04)
ϵ_1	-0.18 (0.32)
γ	-0.08 (0.05)
κ	-0.02 (0.05)
degree	$-3e^{-5}$ ($2.5e^{-5}$)
distance	$-8.6e^{-5}$ ($7.7e^{-4}$)
Infection C * Infection HH	$-5e^{-10}$ ($1.8e^{-9}$)
Infection C * HHCT	$1.3e^{-5}$ ($4.2e^{-6}$)**
Infection C * Community CT	$6.5e^{-6}$ ($4.2e^{-6}$)
HHCT * ϵ_1	-2.21 (0.39)***
Community CT * ϵ_1	-0.34 (0.39)
HHCT * γ	0.21 (0.06)***
Community CT * γ	0.02 (0.06)
HHCT * κ	0.14 (0.06)*
Community CT * κ	-0.01 (0.06)
HHCT * degree	$-5.4e^{-5}$ ($2.8e^{-5}$)
Community CT * degree	$1.8e^{-4}$ ($2.8e^{-5}$)***
HHCT * Distance	$1.5e^{-3}$ ($8.3e^{-4}$)
Community CT * Distance	0.01 ($8.3e^{-4}$)***
Infection C * Infection HH * HHCT	$-5.4e^{-9}$ ($2.2e^{-9}$)*
Infection C * Infection HH * Community CT	$-2.3e^{-9}$ ($2.2e^{-9}$)

$^{***}p < 0.001$; $^{**}p < 0.01$; $^{*}p < 0.05$
