# Supplementary material for: Protective impacts of household-based tuberculosis contact tracing are robust across endemic incidence levels and community contact patterns
Source: PLoS Comput Biol. 2021 Feb 8;17(2):e1008713. doi: 10.1371/journal.pcbi.1008713 (PMC7895355; doi:10.1371/journal.pcbi.1008713)
Supplement: S1 Text — Network kernel formula, generated network features, more details about natural history model and interventions, simulation workflow, epidemiology of modeled population, performance of screening interventions within strata of network parameters and incidence, model trajectories of best performing runs. (PDF) [file pcbi.1008713.s038.pdf]

1 Network connectivity kernel

Network connectivity kernel calculation adapted from Lang et al. [1]. The probability of a connection forming between community contacts is given by:

$$f(d) = (n-4)\frac{e^{(-d^2/2\sigma^2)}/2\pi\sigma^2}{\rho} \quad (1)$$

where $(n-4)$ is the average degree of community contacts. It is offset by 4 which is the number of household contacts. Next, d is the distance between nodes, σ is the average connection radius, and ρ is the density of the nodes (i.e., households) in the network. Individuals must be connected to transmit TB.

2 Features of generated networks

We examined the relationship between average degree, average connection radius, and network clustering coefficient (S1 Fig).

We can see that a higher average connection radius is needed to generate networks with higher average degrees. Furthermore, networks with lower average connection radii and higher average degrees have a higher global clustering coefficient i.e., are more clustered.

The clustering coefficient is calculated by finding all groups of 3 nodes (triads) on a given network with ≥2 ties. Then dividing all triads with 3 ties by the total number triads [2]. A clustering coefficient of 0.4 indicates that 40% of all triads have 3 ties.

3 Natural history model

In our model, individuals are born as susceptible, S and become infected at a rate proportional to the force of infection (FOI) they experience, which consists of household, and community transmission and is defined as:

$$FOI = [\beta_{HH} I_{HH} + \beta_C I_C](1-\omega) \quad (2)$$

where β_{HH} and β_C are the *per-contact* transmission rates of household and community contacts, and I_{HH} and I_C, are the number of household and community contacts with active TB, respectively. Finally, because prior infections confer limited protective immunity [3, 4], the FOI is modulated by ω.

Upon infection, individuals move into the non-infectious early latent state (EL). Within 5 years, ∼13% of individuals in EL progress to infectious active TB (I), with annual rates of progression decreasing with time since infection [5]. These declining rates of progression are represented by 5 distinct EL sub-states (EL_1 to EL_5). After 5 years, the remaining individuals in EL transition to the non-infectious late latent state (LL) with only at most 5% of these individuals developing active TB in their lifetime [6]. See S2 Fig for the distribution of progression times and fraction of individuals progressing across all parameter sets.

Individuals in I may die from TB, spontaneously recover (move to R) [7], or receive treatment (move to T). Additionally, individuals in EL, LL, or R may become reinfected and move back to the EL_1 sub-state. Fig 2 illustrates the disease states and transitions represented in our model.

Individuals may die of either natural causes or TB infection, and upon death are immediately replaced with a susceptible individual. To avoid preferentially placing new susceptible individuals into areas of high TB transmission (which would ultimately overestimate the amount of TB transmission occurring), we implemented a birth reshuffling scheme based on [8] for individuals who die

from TB. In this scheme, individuals who die from TB are replaced with one of their non-infectious contacts (i.e., they could be in any state except for I) who in turn, are replaced with one of their non-infectious contacts and so on until the 3rd replacement is made. Replacements take on the same contact network (i.e. household and community contacts) of those that they replace. Then, a new susceptible individual is born into that 3rd individual's previous location. This shuffling scheme maintains the network structure and a constant number of individuals per household over the course of the simulation.

4 TB screening and treatment

Individuals with active TB (I) who are found through passive case finding are given treatment (moved to the T state). Once in treatment, they are assumed to no longer be infectious and eventually will recover (move to R). Detection of a case through passive surveillance triggers the active case finding (ACF) interventions.

In each screening intervention, contacts found to have TB are placed on treatment (T), while those with latent TB (LTBI) (EL or LL) are given preventive therapy. While undergoing preventive therapy, we assume that individuals cannot be infected or progress to active TB.

5 Comparator active case finding interventions

To determine whether any effects of HHCT could be attributed specifically to focusing ACF at the household level, we made two additional comparisons. First, we implemented community contact tracing (community CT). Here, for each case discovered through passive surveillance, a random subset of 4 community contacts of the index case - equivalent to the number of household contacts - were screened for active TB and LTBI. Second, we included a community-wide ACF scenario. Under this approach, for each case discovered through passive surveillance, 4 individuals were selected at random from the entire population and screened for TB and LTBI (this was adapted from [9, 10]).

In each intervention, up to 4 individuals were followed up and screened for TB immediately following case ascertainment. In the rare case when an individual had <4 community contacts, all of their community contacts were screened. Contacts found to have TB were placed on treatment, while those with LTBI (EL or LL) were given preventive therapy (IPT). While undergoing IPT, we assume that individuals cannot become reinfected or progress to active TB.

6 Simulation strategy

We accounted for parameter uncertainty by running the model with 9,000 parameter sets obtained using Latin Hypercube Sampling [11] from predefined ranges (Table 3). Parameter ranges were either set from the literature (e.g., early latency progression rate), to obtain target incidence levels (e.g., transmission parameters), or to assess the sensitivity of our results to changes in parameter values (e.g., active TB mortality rate). On each simulation run, we selected a network at random so that each network type was run 30 times (there are 300 network types with 10 realizations each). For each parameter set, we ran the transmission model 5 times using a different random seed to allow stochastic variation across realizations.

At the beginning of each run, we seeded the model with a single randomly placed individual with active TB, and ran it (with passive surveillance only) until it reached an steady state. We determined this empirically to be after 6,600 one-month time steps or 550 years. We then began

a simulated trial of ACF for a period of 5 years, representing a plausible time horizon to evaluate the efficacy of an ACF program. We implemented all 3 active case finding scenarios and a passive surveillance only scenario for each parameter set and random number seed combination. This allowed us to directly compare the performance of each intervention by applying it to the same starting conditions at the end of the burn-in period (i.e., right before implementation of ACF). For entire workflow see S3 Fig.

7 Relationship between active TB prevalence and latency levels

Overall, our model reproduced expected behavior associated with different TB incidence levels. Specifically (among passive-detection only runs), the relative distribution of individuals with active disease and latent infection was approximately as expected (S4 Fig). Consistent with Kasaie et al. [10], an active TB prevalence of ∼100 cases per 100,000 person-years conferred a LTBI prevalence of ∼30%.

8 Relationship between community vs. household transmission and incidence

We plotted the relationship between community vs. household transmission (i.e., new EL infections), network clustering coefficient, and incidence levels (immediately before ACF implementation) in our model (S5 Fig). For higher incidence levels, we observe that more infections are caused by community transmission compared with lower incidence levels. Even though the average household transmission rates are higher than the maximum community transmission rates (see Table 3), community transmission is more important in higher incidence settings.

9 Relationship between clustering coefficient and TB incidence

We examined the relationship between network clustering coefficient and incidence immediately before ACF (S6 Fig).

As clustering coefficient increases, incidence levels (immediately before ACF) decrease. This is likely due to contact saturation in highly clustered networks.

10 Relationship between network parameters, transmission rates, and incidence

We examined the relationship between network parameters (i.e., average degree, average connection radius) and transmission rates colored by incidence levels immediately before ACF (S7 Fig).

We observed that while higher household and community transmission rates lead to increased incidence levels, there are interactive relationships between average degree, average connection radius, and transmission rates. For instance, the same incidence level can be achieved with different combinations of average degree and per-contact infectiousness. A higher average degree can tradeoff with a lower community transmission rate to obtain a given incidence level.

11 Performance of HHCT within strata of network parameters and incidence

We examined how the effectiveness of HHCT varies in response to different (pre-ACF) incidence levels or contact patterns using passive-detection only as a reference group (S1-S4 Tables and S8 Fig).

12 Ridgeline plots showing performance of screening interventions within strata of incidence level and network parameter

We examined how HHCT performed within pairwise distributions of incidence-level and network parameter strata e.g., incidence levels by average degree (S9-S12 Figs).

13 Best performing model runs among incidence levels between 100 and 200 cases per 100,000 person-years

We plotted the 100 best performing model runs (i.e., with the lowest rate ratios) for HHCT among incidence levels between 100 to 200 cases per 100,000 person years. We can see here the immediate and dramatic effect of HHCT has on TB prevalence (S13 Fig).

14 Comparison of effort per contact tracing effort: HHCT vs. community CT

We examined how the effectiveness of community CT varies in response to different incidence levels or community contact patterns using passive surveillance only as a reference group.

Although HHCT conferred similar protective benefits across all network settings (e.g., when varying average degree or average connection radius), community CT appeared to be more sensitive to changes in community contact patterns (S5-S8 Tables and S14 Fig). Notably, the effect of incidence level on community CT was similar to HHCT. Specifically, as average degree and average connection radius increased, community CT became slightly less effective. Additionally, due to overlapping contacts being able to sustain transmission in the community, community CT was slightly more effective as network clustering coefficient increased. In other words, in more clustered networks, community CT is likely to more effectively target the source of transmission to index cases i.e., community contacts. However, the relationship between clustering and community CT is complex. Importantly, these emergent trends only resulted in slight changes to the effectiveness of community CT with the standard deviations associated with the RRs being greater than the differences between strata.

The fact that community CT was sensitive to different network parameters is likely in part due to the limited reach of our modeled community CT scenario, since only a small fraction of community contacts of a given index case were screened for TB (on average the maximum being $\sim$20%.

15 Comparison of effort per screening effort: HHCT vs. community-wide ACF

We examined how the effectiveness of community-wide ACF varies in response to different incidence levels or contact patterns using passive surveillance only as a reference group (S9-S12 Tables and S15 Fig).

16 Mean Infectious Period Duration by Intervention

We examined how the mean infectious period of individuals with active TB varies by intervention (S16 Fig).

17 Secondary Cases Averted Among Household Contacts

We compared the approximate number of secondary cases averted by preventive therapy among household contacts (S17 Fig). To do this, we calculated a household level R_0:

$$R0_{HH} = contacts(1 - e^{\frac{-\beta_{HH}}{\gamma + CDR + \kappa + \frac{1}{\theta}} PR(progression)}) \tag{3}$$

where contacts are the number of household contacts (we assume all contacts are susceptible), β_{HH} is the household transmission rate, γ is the spontaneous recovery rate of individuals with active TB, CDR is the rate at which individuals with active TB self-present for care, κ is the active TB mortality rate, and θ is the life expectancy. The parameters in the denominator accounted for competing risks which alter the duration of the infectious period. We also accounted for the probability of progression 'PR(progression)' to active disease among infected individuals. Overall, we calculated the household level R_0 among all parameter sets and found that an infectious case in a fully susceptible household will cause a median of ~ 0.31 active TB cases. Therefore, we multiplied the number of preventive therapy administrations in each screening scenario by 0.31.

18 Number of Preventive Therapy and Treatment Administrations by Intervention

For each model run, we calculated the number preventive therapy administrations (given to individuals with LTBI) divided by treatment administrations (to individuals with active TB) of LTBI and active TB by screening intervention (S18 Fig).

19 Prevalence of Latent and Active TB Among Household and Community Contacts

We calculated the prevalence of LTBI and active TB among household and community contacts by screening intervention (S19 Fig).

20 HHCT Perfomance by Community and Household Transmission

We examined how the impact of HHCT changes when varying the relative contribution of community to household transmission (S20 Fig).

We note that when there is more household transmission than community transmission, HHCT performs slightly better. However, overall the performance of HHCT is largely the same across settings.

21 Varying HHCT Coverage

We conducted a sensitivity analysis to examine the effects of varying HHCT coverage. Specifically, for each parameter set, random seed, and intervention combination, we varied the percentage of index case households that we screened by randomly sampling a level of coverage between 0% and 100%. We examined the results within strata of pre-intervention incidence (S21 Fig).

22 Examining Source of Variability in Model Outputs

We constructed a hierarchical model to examine the relative contribution of stochasticity, networks and model parameter values to the overall variability in rate ratios in the model results:

$$RR = \beta_1 \frac{Infection_C}{Infection_H H} S + \beta_2 \epsilon_1 S + \beta_3 \gamma S + \beta_4 \kappa S + \beta_5 degree S + \beta_5 distance S + bNetwork \tag{4}$$

where the outcome are rate ratios (RR) comparing the cumulative incidence at the end of the intervention to the passive surveillance only, β's are fixed effect coefficients and b is the random effects coefficient. $Infection_C$ and $Infection_H H$ are infections attributable to the community and household, respectively. Next, 'S' is screening scenario, ϵ_1 is the rate of progression to active TB in the first year of infection, γ and κ are the recovery rate and mortality rate among individuals with active TB, respectively. Finally, 'degree' is the average degree 'distance' is average connection radius of the network (S13 Table).

Overall, we found that the model $R^2 \sim 85.6\%$. The variability in RR estimates due to stochasticity was $\sim 5\%$ and the variability due to network realization was $\sim 3.5\%$. Therefore the parameterization of the model was responsible for the majority of variability observed in rate ratios.

23 Impacts of Incorporating Imported TB Cases

To explore the how imported active TB cases alter the impacts of interventions, we conducted a sensitivity analysis in which we assumed between 2 to 20 cases of TB were imported per month.

We incorporated this additional risk into the force of infection as a background rate by scaling β_C by the total population and multiplying that by the number of imported TB cases per month (we randomly sampled a value between 2 and 20). Therefore the force of infection for this sensitivity analysis was as follows:

$$FOI = [\beta_{HH} I_{HH} + \beta_C I_C + \beta_M I_M](1 - \omega) \tag{5}$$

consistent with the derivation of β_C, we calculate β_M by multiplying β_{HH} by a scaling factor between 0 and 1 and then dividing by the number of individuals at risk (which in this case is the entire population).

Overall, we found that general conclusions did not change. We did however find that the impacts of all interventions were reduced. Additionally, interventions had substantially lower impacts at

lower incidence levels because the effects of imported cases contributed to most of the overall TB burden and the interventions did not target imported cases (S22-S24 Figs).

References

[1] Lang JC, De Sterck H, Kaiser JL, Miller JC. Analytic models for SIR disease spread on random spatial networks. Journal of Complex Networks. 2018;6(6):948–970.

[2] Wasserman S, Faust K, et al. Social network analysis: Methods and applications. vol. 8. Cambridge university press; 1994.

[3] Woldehanna S, Volmink J. Treatment of latent tuberculosis infection in HIV infected persons. The Cochrane database of systematic reviews. 2004;(1):CD000171–CD000171.

[4] Andrews JR, Noubary F, Walensky RP, Cerda R, Losina E, Horsburgh CR. Risk of progression to active tuberculosis following reinfection with Mycobacterium tuberculosis. Clinical infectious diseases. 2012;54(6):784–791.

[5] Vynnycky E, Fine P. The natural history of tuberculosis: the implications of age-dependent risks of disease and the role of reinfection. Epidemiology & Infection. 1997;119(2):183–201.

[6] Horsburgh Jr CR. Priorities for the treatment of latent tuberculosis infection in the United States. New England Journal of Medicine. 2004;350(20):2060–2067.

[7] Tiemersma EW, van der Werf MJ, Borgdorff MW, Williams BG, Nagelkerke NJ. Natural history of tuberculosis: duration and fatality of untreated pulmonary tuberculosis in HIV negative patients: a systematic review. PloS one. 2011;6(4):e17601.

[8] Cohen T, Colijn C, Finklea B, Murray M. Exogenous re-infection and the dynamics of tuberculosis epidemics: local effects in a network model of transmission. Journal of the Royal Society Interface. 2006;4(14):523–531.

[9] Zelner J, Murray M, Becerra M, Galea J, Lecca L, Calderon R, et al. Protective effects of household-based TB interventions are robust to neighbourhood-level variation in exposure risk in Lima, Peru: a model-based analysis. International journal of epidemiology. 2017;.

[10] Kasaie P, Andrews JR, Kelton WD, Dowdy DW. Timing of tuberculosis transmission and the impact of household contact tracing. An agent-based simulation model. American journal of respiratory and critical care medicine. 2014;189(7):845–852.

[11] Stein M. Large sample properties of simulations using Latin hypercube sampling. Technometrics. 1987;29(2):143–151.
