# Supplementary material for: Protective impacts of household-based tuberculosis contact tracing are robust across endemic incidence levels and community contact patterns
Source: PLoS Comput Biol. 2021 Feb 8;17(2):e1008713. doi: 10.1371/journal.pcbi.1008713 (PMC7895355; doi:10.1371/journal.pcbi.1008713)
Supplement: Attachment — Submitted filename: reviewer_response_letter.pdf [file pcbi.1008713.s039.pdf]

December 2nd, 2020

Dear Editors of *PLOS Computational Biology*:

We appreciate the opportunity to submit a revised version of our manuscript “Protective impacts of household-based tuberculosis contact tracing are robust across endemic incidence levels and community contact patterns”.

We would like to thank the editor and reviewers for their helpful comments and suggestions. We have re-ran the analysis, regenerated all figures and tables and have added additional analyses and text throughout the manuscript. Point-by-point responses to all editor and reviewer comments are below.

Thank you again for your consideration of our revised manuscript.

Sincerely,

Joshua Havumaki
Yale University

Editor Comments:

In addition to the reviewers' comments, I have two suggestions:

a) It would be good if the authors could also publish the model code (e.g. on github) to ensure reproducibility.

> **Response**: We thank the editor for their suggestions. We have added the model code to GitHub: https://github.com/jhavumaki/network_tb_ibm and have referenced it in the "simulation strategy" section of the "methods".

b) It would be interesting to use the model to explore alternative explanations for the mixed results of trials on HHCT (e.g. a more formal exploration of the hypothesis given in the discussion "other potential explanations for the mixed results of trials of HHCT, including the coverage and quality of case finding activities, probably are more responsible for variable results than the underlying epidemiological situation. " and/or other explanations)

> **Response:** Thank you for your comment. This is a good point. We have conducted a sensitivity analysis examining how impacts change when varying HHCT coverage. We found that among higher incidence level settings >100 cases per 100,000 person-years, low HHCT coverage can result in negligible protective impacts (i.e., rate ratios near 1). We have added text to the results and discussion and a figure to the "Supporting Information" section.

Reviewer #1 Comments

Reviewer #1: Havumaki et al developed a spatial model of TB transmission to understand the impact of Household contact tracing on transmission. The main justification for this undertaking are mixed results from RCTs (i.e., the Zamstar trial). The main finding is that in this model, HHCT is robust across all epidemiological setting, even in high transmission setting with community transmission. In contrast, community contact tracing appeared to be more sensitive to differences in the network model. The model relies on oversimplifications (adressed in the discussion) which are necessary for the analysis but impair generalization/translation into clinics.

Overall, I think it is an intriguing hypothesis that HHCT remains effective in settings of high community transmission. This is a well written manuscript. The main weakness of the paper is the fact that the authors rely solely on their in silico model.

Thus far, the authors focused mainly on high transmission settings. I think it would be very interesting to apply the model to low transmission settings in a more detailed way. A considerable effort is being undertaken by authorities in low transmission countries to track mostly imported cases both in the house hold contact setting as well as the community. To me, it would be very interesting to use the model to see how effective HHCT and the other tracking methods are (for example in expat communities or refugee camps) under the assumption that a certain number of new cases is imported on a regular basis from highly mobile individuals.

<u>Response</u>: We thank reviewer #1 for their helpful comments. We decided to motivate our analysis based on the assumption that although HHCT is already conducted in low incidence settings, there is an implication that HHCT will not be efficient in higher incidence settings due to genotypic studies which have found that the majority of transmission occurs in the community in high burden settings [1]. Therefore, we decided to explore impacts of HHCT in both moderate [2] and higher incidence settings where there is sustained endemic community transmission. Despite the implication from genotypic studies, our findings suggest that HHCT is actually effective in higher incidence settings regardless of contact patterns or incidence level. We have added clarifying text to the abstract, author summary, and the model parameters section of the methods.

To explore the effects of imported cases, we conducted a sensitivity analysis in which 2 to 20 cases of TB are imported per month. The results of this analysis show that imported cases reduced the impacts of all screening interventions. However overall, we found that the qualitative results and general conclusions of our analysis did not change. We added text to the "Results" and figures to the "Supporting Information".

References:

1. McCreesh, Nicky, and Richard G. White. "An explanation for the low proportion of tuberculosis that results from transmission between household and known social contacts." Scientific reports 8.1 (2018): 5382.
2. World Health Organization. "Global tuberculosis report 2019. 2019." Geneva: World Health Organization (2019).

I don`t have the enough networking modeling experience to judge whether the methods employed are adequate and I recommend statistical review.

Reviewer #2 Comments

Reviewer #2: This article considers the transmission epidemiological features that might be expected to influence the relative impact of household contact tracing and preventive therapy on tuberculosis incidence. Their main conclusion is that the impact of this strategy is robust across epidemic intensities, which is interesting and important in my view.

<u>Response</u>: we thank reviewer #2 for their helpful comments.

Given the nature of this result, and the stochasticity involved in results and number of things varying, the main point I would seek reassurance around it that the experimental design used is adequate in terms of: a) choice of metrics used to characterise epidemiology; b) power to detect trends in outcomes with respect to these metrics. Separately, I thought it strange not to quantify the impact achieved via early detection of cases vs PT, and whether this varied by intervention strategy.

<u>Response</u>: Thank you for your comment. We agree that these are important considerations. Here is how we accounted for these factors:

a. To characterize the epidemiology of our modeled population, we examined all model runs in the absence of active case finding. Specifically, we found that the proportion

of individuals with latent TB was approximately as expected for different TB prevalence levels [1]. Additionally, consistent with genotypic data from high burden settings [2], we found that a greater proportion of community infections were caused by community transmission in higher incidence settings.

b. Our general conclusions are based on overall trends in the model results. For instance, in the spline plots (Fig 3), we found that the performance of HHCT is largely consistent for all incidence and network parameter values with all mean rate ratios being approximately 0.72 (Tables S1 to S4). In other words, we are not focusing on every minor change in the splines (Fig 3). Therefore, we believe that there is sufficient power to detect trends in intervention outcomes. To further address this and account for stochasticity, we are now presenting intervention results using the 5-year cumulative incidence (instead of the incidence in the final year of the intervention). This has made the trends supporting our general conclusions more apparent.
c. Our model does not explicitly simulate individuals infecting other individuals (it is based on weighted number draws). Therefore, it is difficult to quantify the actual impact achieved by treatment vs. preventive therapy. To get a sense of the relative number of preventive therapy administrations compared with treatment administrations, we added a ridgeline plot displaying the ratio of preventive therapy administrations to treatment administrations by screening scenario to the Supporting Information. We found that HHCT resulted in substantially more preventive therapy administrations which implies that it was a more effective, targeted approach for curbing transmission and targeting individuals at risk for progressing to infectious TB.

References:

1. Houben, Rein MGJ, and Peter J. Dodd. "The global burden of latent tuberculosis infection: a re-estimation using mathematical modelling." PLoS medicine 13.10 (2016): e1002152.
2. Verver, Suzanne, et al. "Transmission of tuberculosis in a high incidence urban community in South Africa." International journal of epidemiology 33.2 (2004): 351-357.

I appreciate these data may have been computationally onerous to generate (although this is not reported on), but I hope some of the below might be possible to explore by analysing the data that already exist.

Since there are three sources of variation in outputs (parameters, networks, intrinsic stochasticity), I would have found it useful to get a sense of which contributed most to overall variation, and how large the variation in impact was.

<u>Response</u>: Thank you for your comment. To address this, we constructed a hierarchical model with the rate ratios (comparing screening interventions to passive surveillance) as the outcome, the parameterization of the model as fixed effects and network realizations as random effects. We used this model to assess the relative contribution of each of these components to the R^2 value. Overall, we found that the network realization and stochasticity contributed substantially less than the parameterization of the model to overall variation in rate ratios. We have

added a paragraph to the results to describe these findings and a section to the Supporting Information.

The authors used the 5-year incidence end-point ratio as outcome I believe; I wonder whether they explored cumulative incidence as an outcome metric (smaller mean effect but smaller stochasticity)?

Response: thank you for this suggestion. This is a good idea. We generated the results using the 5-year cumulative incidence (instead of the annual incidence at the end of the intervention) and found that they are clearer and more straightforward to interpret. We have therefore replaced all results with 5-year cumulative incidence as the outcome metric. Our overall conclusions have not changed.

In terms of metrics that may correlate with impact, I wondered about other possibilities than incidence and network features.

Response: To address this, we have added the hierarchical model to the Supporting Information which shows how each parameter affects the overall impact of screening scenarios (i.e., rate ratios). Furthermore, we examined how the proportion of community vs. household transmission affects the performance of HHCT and have added a figure illustrating this relationship to the Supporting Information.

From figure S4, there seems little relation between incidence and proportion of incidence due to household transmission. I wonder whether the proportion of transmission within the household explains more variation in the impact of HHCT?

Response: Thank you. We have added a figure to the Supporting Information which explores the relationship between community vs. household transmission and the performance of HHCT. Overall, although there are small changes when household transmission is higher than community transmission, the impacts of HHCT are largely consistent.

Was the time-since-incidence recorded at detection recorded? I was curious whether there were differences in the mean time as a prevalent case, and the mean number of secondary infections generated by time of detection for cases found via HHCT and via other means.

Response: Thank you for your comment. We have added ridgeline plots showing the mean time as a prevalent case and approximate number of secondary cases averted by preventive therapy for each screening scenario. Because transmission in our model occurs through weighted random number draws, it does not explicitly simulate individuals infecting other individuals. Instead an infectious individual contributes to their contacts' force of infection which increases the risk of their contact becoming infected. Therefore, to approximate the number of secondary infections averted, we formulated a household R_0 which is derived based on the household transmission parameter, duration of infectiousness and the probability of progression. We found that over all parameter sets, a given infectious individual will cause a median of 0.31 additional cases in their household (if all their contacts are susceptible). This calculation problem becomes very quickly intractable when incorporating community contacts, therefore we used the

household R_0 as a rough proxy to estimate secondary cases averted. Furthermore, we only compared preventive therapy administrations as a rough proxy as well.

We have added an explanation and figures to the Supporting Information and mention this analysis in the results section.

In a similar vein, it would be interesting to know the typical prevalence among HH vs community contacts (both active TB and LTBI).

Response: Thank you for your comment. We have added ridgeline plots showing the distributions of latent TB and active TB prevalence among household and community contacts to the Supporting Information and mention this analysis in results section.

I think the discussion of limitations is quite focussed on technical aspects within the model universe. Heterogeneity gets a mention in future work, but perhaps clustering of tuberculosis due to shared risk factors (rather than transmission) and changes in infectiousness over time should get mentions as potentially important aspects of reality for this question that were not modelled.

Response: Thank you for your comment. This is a good point and we have added text to the limitations section of the discussion. We agree that future models exploring the impact of individual-level heterogeneity on the robustness of HHCT will help clarify the real-world utility of this approach. With respect to changes in infectiousness over time, we believe that incorporating this would not have differentially affected the performance of interventions and therefore our conclusions would not have changed.

Implementation details. One gathers in various places that a time-step of 1 month is used, but otherwise no details of the transmission model implementation are given, or links to source code. I think there should at least be a few sentences somewhere explaining what kind of simulation approach was used, programming language used, some sense of compute effort for results.

Response: Thank you for your comment. We have added implementation details to the Simulation strategy section in the methods.

more minor points

- 'cdr': as a parameter name and description as 'case detection rate' might be confusing given that it does not correspond to the tuberculosis conventional case detection rate (notifications over incidence in a year)

Response: Thank you, we updated the definition in Table 2 to "The rate at which individuals with active TB self-present for care".

- theta: in the parameter table this is given as a life expectancy, but I think it is the reciprocal of this as used in the model

Response: we have updated the definition in Table 2.

- EL progression structure: what was the rationale for including the 5 state progression? The implied fraction progressing was stated informally (but I think differently) in the supplement and text. I would find it helpful to see in the supplement the associated

survival function for the progression model (or distribution of times to progression) and actual fraction progressing (calculated for a cohort; not from the agent based model). Perhaps this could be done along with calculating mean number of secondary infections upon detection.

> **Response**: thank you for your comment. We chose the 5-state progression structure to capture the decreasing rate of progression since time of infection (as described in Figure 2 of [1]). To further characterize this structure, we have calculated the fraction progressing to active TB for all parameter sets for (1) individuals in the early latent state (i.e., 5-years since infection) and (2) overall, for individuals in both early and late latent states. We have also taken the mean estimate for early latent progression (across all parameter sets) and the late latent progression rate and have simulated the distribution of progression times for early latent and overall. We have added both figures to the Supporting Information.
>
> References:
>
> 1. Vynnycky, E., and P. E. M. Fine. "The natural history of tuberculosis: the implications of age-dependent risks of disease and the role of reinfection." Epidemiology & Infection 119.2 (1997): 183-201.

- Figure S14: why does this begin above 1?

> **Response:** This is no longer the case after changing the outcome metric to the 5-year cumulative incidence. This was due to stochasticity and small differences between the null and maximum rate ratio i.e., it was a difference of 1 or 2 cases.

- 'equilibrium': to be pedantic, the only equilibrium for a stochastic model like this is the disease free one after extinction.

> **Response:** we have changed "equilibrium" to "steady state"

- outlier exclusion: The figures contain a sentence (although it doesn't have a verb) beginning 'Among..' about excluding the 5% most extreme results. I think in the supplement somewhere this is described as outlier exclusion. First, I think this needs to be in the main article methods (sorry if I missed). Secondly, could more be presented about how much difference this makes and what is going on with the excluded runs?

> **Response**: thank you. We have replaced all results in the main paper and now include all model runs. This did not change our overall conclusions.

- ACF: I think this makes first appearance in a section heading without previously being defined?

> **Response**: we are no longer abbreviating ACF in the section heading.

- spatial extent: I don't remember reading the dimensions of the spatial extent. I suppose it is implied by the houshold density; but could you state it please in methods? This would make it easier to interpret the average connection radius parameter (sigma in table 1).

> **Response:** thank you. The spatial extent is indeed implied by the network density which we fixed to 1. The 'x' and 'y' coordinates of the grid varied from 0 to sqrt(population_size/household_size). In other words, each household was placed

randomly in a 2-dimensional space of normalized area equal to the total number of households. We have added details to the spatial contact network section in the methods.

Reviewer #3 Comments

Reviewer #3: Major Comments:

1. I found the description of the model a bit hard to follow, especially the spatial contact network part. Since the model is quite complex, and tries to bring different ideas and modeling techniques, the authors could be more thorough and careful with their description of the model.

See some specific comments below.

Response: we thank reviewer #3 for their helpful comments.

2. The results/findings are underwhelming and difficult to make sense, especially given that authors have put together a complex model to explore these phenomena.

E.g., the authors find that effect of HHCT is on average similar across low and high-incidence settings. However, low and high incidence settings can be conceived with various parameter combinations (as seen in Fig S-6), given a flexible model structure.

Hence, the relationship would really only reflect the conceptualization of low and high incidence settings, and it is unclear why one should expect the impact to vary significantly.

Response: thank you for your comment. In addition to incidence level, we have also included different network types representing social mixing patterns (e.g., many connections vs. few connections or disperse vs. localized transmission) in our definition of setting. We have clarified the wording throughout the text to emphasize this point. Overall, we found that HHCT is also robust across different network settings (in addition to different incidence levels). We understand that the results were not completely clear and readily interpretable given the complexity of the model and have therefore constructed a hierarchical model which quantifies the relative contribution of stochasticity, network realizations and parameters in the model to the overall outcome metrics (i.e., rate ratios). We found that parameterization of the model contributes substantially more to the impact of screening scenarios than the network realization and stochasticity. Additionally, to further compare across settings, we examined how the proportion of community vs. household transmission might affect HHCT performance. This allowed us to directly examine our hypothesis i.e., the relative concentration of household vs. community transmission may alter the impacts of HHCT. However, consistent with the rest of our analysis, HHCT appeared to be robust across different transmission settings. We have added these analyses to the Supporting Information.

References:

1. McCreesh, Nicky, and Richard G. White. "An explanation for the low proportion of tuberculosis that results from transmission between household and known social contacts." Scientific reports 8.1 (2018): 5382.

Perhaps focusing on specific conceptualization of the models (e.g. low incidence, with 20% HH transmission vs 50% HH transmission, or low and high incidence settings with 20% HH transmission), would help

illustrate how impact of HHCT could vary/not vary by the incidence of the setting, or by the relative role of community transmission. As it stands, it is hard to know what to make of the results because they aggregate over various scenarios.

Also, I am not convinced with the authors' conclusion that CCT are different from HHCT (see below).

> **Response**: thank you for your comment. To further elucidate this, we examined how the proportion of community vs. household transmission might affect HHCT performance (Supporting Information). Consistent with other results, HHCT was found to be robust across different transmission settings. Additionally, we believe that the distinction between HHCT and community CT is even more evident across different network types using the 5-year cumulative incidence outcome metric (we were previously using the incidence from the final year of the intervention). We have replaced all results using the 5-year cumulative incidence.

Minor Comments:

Abstract:

Not sure if I follow the logic, why should individuals preferentially mix more locally in low burden settings compared to higher burden setting?

> **Response:** Thank you, we agree this was not clear and have clarified the wording. We are describing settings that are (1) low incidence and (2) in which individuals preferentially mix locally. We are not trying to infer that individuals mix more locally in low burden settings.

Introduction

lines 28-29: Perhaps useful to elaborate what authors mean by social mixing patterns.

Also, in the following sentence "..longer distance between community contacts", it is unclear what distance the authors are referring to, physical distance vs distance in the contact network.

> **Response:** Thank you, we have clarified the wording here to indicate that social mixing patterns refer to the average number of contacts and physical distance between contacts.

Methods:

lines 39-41: It would be helpful for the readers if some of the key features of the two previously published models that the authors are extending in this analysis are elaborated.

> **Response:** we have added more details to the methods section.

Household size is fixed at 5 for all households.

line 48: I think it would be useful to describe terms such as contacts, average connection radius in the context of TB transmission dynamics. eg, should a contact thought of as transmission link, potential transmission link, or just individuals that come in to contact for some duration/frequency?

Response: Thank you, we agree this was not clear from the description in the text. We have updated the text in the spatial contact network section of the methods to describe what contacts represent (i.e., potential transmission links with household contacts being close contacts and community contacts being casual contacts). We also explain how varying the average degree and average connection radius might alter transmission across the network.

Line 60: Describe, and provide equation for global clustering coefficient, C. It is unclear how exactly clustering coefficient is calculated.

Response: We have added text to the methods section to include more details about how we calculated the clustering coefficient. We assumed that each household is a node and calculated the clustering coefficient by finding triangles in the network i.e., groups of 3 households that are connected by either 2 or 3 connections (2 households were connected if they had at least one community connection between them). The clustering coefficient is then equal to the number of closed triangles (i.e., groups in which all households are connected to each other) over the total number of triangles.

Table 2: I find it hard to believe that variance in HH transmission is the primary driver of difference in TB incidence. Differences in progression during latency, time to diagnosis, and transmission in community are important.

Response: thank you for your comment. We agree that these factors are important drivers of TB incidence. Our analysis primarily focused on how variation in the proportion of household vs. community transmission alters the impacts of HHCT. Therefore, we allowed for large ranges in the community and household transmission parameters. We also varied the rate of progression, but in our model, the household and community transmission rates had the largest effects on incidence. We have clarified the focus of our analysis in the model parameters section of the methods. We also note in Table 2 that the community transmission parameter has large effects on overall incidence. Progression during latency and time to diagnosis have important implications for heterogeneity on the individual level, but incorporating a more detailed representation of these factors was beyond the scope of our analysis.

Results:

lines 162-163: The ratio of infection in the community to HH in the simulations range between 0.5-3, but studies find only 10-20% of infections occur in HH (ref 30). It may make sense to

explore a range that is closer to these data.

Response: thank you for your comment. While these studies serve as an important motivation for our analysis, they only provide data from households with co-

prevalent cases. On the other hand, results from our model display the ratio of infections (not only cases) attributable to community vs. household transmission across all households (i.e., not only ones with co-prevalent cases). Therefore, we cannot make a direct comparison. We do however note that (qualitatively consistent with the literature) as incidence level increases, more infections are attributable to the community.

Fig 4: The correlation between CCT and average degree is to be expected.

For a given level of incidence, lower average degree would imply higher transmission between community contacts, all else being equal.

Which in turn would mean that screening fixed number of those contacts are more effective in detecting TB infection and disease.

However, the CCT does not differ much by incidence, Fig S-13 (top-left), same as for HHCT.

Hence, I am not sure that HHCT and CCT differ much by setting. I would also suggest the authors to include Fig S-13 instead of 4.

Response: thank you for your comment. We have also included different community transmission patterns or network types (e.g., many connections vs. few connections or disperse vs. localized transmission) in our definition of setting. We clarified the wording throughout the text to emphasize this point. Although community CT does not differ by incidence level setting, it does when we vary community contact patterns.
