# Supplementary material for: Protective impacts of household-based tuberculosis contact tracing are robust across endemic incidence levels and community contact patterns
Source: PLoS Comput Biol. 2021 Feb 8;17(2):e1008713. doi: 10.1371/journal.pcbi.1008713 (PMC7895355; doi:10.1371/journal.pcbi.1008713)
Supplement: Attachment — Submitted filename: response_to_reviewer_210107.pdf [file pcbi.1008713.s040.pdf]

January 7th, 2020

Dear Editors of *PLOS Computational Biology*:

We once again appreciate the opportunity to submit a revised version of our manuscript "Protective impacts of household-based tuberculosis contact tracing are robust across endemic incidence levels and community contact patterns".

We would like to thank the editor and reviewers for their helpful comments and suggestions. We have added text throughout the manuscript with point-by-point responses to the reviewer comments below.

Thank you again for your consideration of our revised manuscript.

Sincerely,

Joshua Havumaki
Yale University

Reviewer #4: The authors have answered all of the reviewers answers and questions. They also added some material in the manuscript and in the SI, and the additional results and sensitivity analyses are consistent with the main results. As highlighted by R1, the main weakness of the paper is the lack of application to real data. I have some additional remarks in that sense:

- the authors focused their analysis on moderate and high incidence settings (to explore the effect of HHCT in these specific cases). I think it would be useful to give some incidence values in some moderate/high incidence areas, to have an idea of where those areas/countries would compare to the model simulations

> **Response:** we thank reviewer #4 for their comments. We have rephrased the wording in the model parameters section and have included example countries which have estimated incidence levels that cover this range.

- I understand that a good amount of sensitivity analyses were already performed, but I wonder to which extend considering a more heterogeneous repartition of the population (variable number of individuals per househould, not a uniform repartition on the grid) would change (or not) the results.

> **Response:** Thank you, we agree that this is a potentially interesting area of additional investigation. While it is not computationally possible to explore local variation in household size in the context of our existing model, we note that the effects of HHCT have been found to be robust to observed variation in local community-level exposure in one setting [1]. We have updated the discussion (limitations and future directions) to mention this.

> References:

1. Zelner, Jon, et al. "Protective effects of household-based TB interventions are robust to neighbourhood-level variation in exposure risk in Lima, Peru: a model-based analysis." International journal of epidemiology 47.1 (2018): 185-192.

- line 127: the authors constrained the rate at which individual with active TB seek care (average of 1 year). How realistic is this 1 year value?

> **Response:** Assuming that prevalence = incidence x duration, we can approximate the global duration of TB disease using the estimates from [1]. If there are an estimated 11 to 13 million prevalence cases of TB and 8.3 to 9 million incident cases, then the duration of active disease is ~1 year. This duration of active disease is consistent with most similar models of TB dynamics in the literature, and other analyses. We have updated Table 2 to reflect this.

> References:

1. Glaziou, Philippe, et al. "Global epidemiology of tuberculosis." Cold Spring Harbor perspectives in medicine 5.2 (2015): a017798.

- the authors found that HHCT resulted in lower prevalence of active TB among both households and community contacts, but the prevalence of LTBI was similar between HHCT and community CT. How can we explain that the prevalence of LTBI was not reduced by the HHCT, and which parameter in the model (i.e., which additional intervention in the HHCT) could help reducing this prevalence?

> **Response:** Thank you. The prevalence of LTBI represents a cumulative lifetime exposure status and therefore changes much more slowly than the prevalence active TB. We only ran the intervention for 5 years and therefore did not see substantial effects on the LTBI prevalence which will only be expected to change on demographic timescales. We have updated the Comparator Interventions section of the results to clarify.
